# Role of fibulin-5 insufficiency and prolapse progression on murine vaginal biomechanical function

**DOI:** 10.1038/s41598-021-00351-1

**Published:** 2021-10-25

**Authors:** Gabrielle L. Clark-Patterson, Sambit Roy, Laurephile Desrosiers, Leise R. Knoepp, Aritro Sen, Kristin S. Miller

**Affiliations:** 1 Department of Biomedical Engineering, Tulane University, New Orleans, 70118 USA; 2 Department of Animal Sciences, Reproductive and Developmental Sciences Program, Michigan State University, East Lansing, 48824 USA; 3 Department of Female Pelvic Medicine and Reconstructive Surgery, University of Queensland Ochsner Clinical School, New Orleans, 70121 USA

**Keywords:** Biomedical engineering, Urogenital reproductive disorders

## Abstract

The vagina plays a critical role in supporting the pelvic organs and loss of support leads to pelvic organ prolapse. It is unknown what microstructural changes influence prolapse progression nor how decreased elastic fibers contributes to vaginal remodeling and smooth muscle contractility. The objective for this study was to evaluate the effect of fibulin-5 haploinsufficiency, and deficiency with progressive prolapse on the biaxial contractile and biomechanical function of the murine vagina. Vaginas from wildtype (n = 13), haploinsufficient (n = 13), and deficient mice with grade 1 (n = 9) and grade 2 or 3 (n = 9) prolapse were explanted for biaxial contractile and biomechanical testing. Multiaxial histology (n = 3/group) evaluated elastic and collagen fiber microstructure. Western blotting quantified protein expression (n = 6/group). A one-way ANOVA or Kruskal–Wallis test evaluated statistical significance. Pearson’s or Spearman’s test determined correlations with prolapse grade. Axial contractility decreased with fibulin-5 deficiency and POP (p < 0.001), negatively correlated with prolapse grade (ρ = − 0.80; p < 0.001), and positively correlated with muscularis elastin area fraction (ρ = − 0.78; p = 0.004). Circumferential (ρ = 0.71; p < 0.001) and axial (ρ = 0.69; p < 0.001) vaginal wall stresses positively correlated with prolapse grade. These findings demonstrated that fibulin-5 deficiency and prolapse progression decreased vaginal contractility and increased vaginal wall stress. Future work is needed to better understand the processes that contribute to prolapse progression in order to guide diagnostic, preventative, and treatment strategies.

## Introduction

Pelvic organ prolapse (POP) is defined as anatomical changes due to falling, slipping or downward displacement of the uterus or vaginal compartments and their neighboring organs (bladder, rectum, or bowel)^1^. POP symptoms can include vaginal bulge, pelvic pressure, and is often associated with other pelvic floor disorders, like urinary and fecal incontinence^2^. Conservative, nonsurgical, options such as physical therapy may be prescribed to patients as a first-line option to reduce symptoms, prevent POP progression, and to avoid or delay the need for surgery^3,4^. However, these interventions are usually most beneficial for patients with mild to moderate POP^3,4^. 12.6% of women will undergo surgery for POP in their lifetime^5^. POP reoccurrence rates are up to 38% with 3% undergoing repeated operation within 3 years, and 12% present with complications due mesh exposure^6^. While vaginal birth and aging are well established risk factors for POP, the underlying mechanisms that contribute to the development and progression of severe POP are poorly understood. Damage to the levator ani muscle during vaginal birth is highly implicated, however, not all women with levator ani tears develop POP and not all women with POP have levator ani tears^7^. Therefore, the underlying processes of POP are more likely multifactorial and recent studies suggest that vaginal connective tissue dysfunction due to elastic fiber disruption may contribute to the development and progression of POP^8–11^.

Elastic fibers consist of various structural components (e.g., elastin, fibrillins, and fibulins) that are important for the assembly into a mature and functional fiber. Elastic fibers contribute to the integrity of the vaginal wall by imparting compliance and extensibility^12–14^. Additionally, intact and mature elastic fibers may be critical to maintain smooth muscle cell (SMC) contractility^15–18^. POP is common in women with certain, genetic disorders, such as Marfan syndrome, which results in defects in elastic fiber synthesis or assembly^11^. A decrease in elastic fiber components, elastin and fibulin-5, in prolapsed human vaginal tissue correlates with POP severity^19–22^. Further, women with POP have stiffer vaginal tissue, which correlates with POP severity, compared to non-POP controls^23–27^.

Currently it is not known what microstructural changes influence POP progression, nor how decreased elastic fibers contribute to vaginal remodeling and SMC contractility. Although not a substitute for human tissue, mouse models are useful for investigating protein and cellular interactions to elucidate the underlying biological processes and pathologies. By 6 months of age 92% of fibulin-5 deficient mice develop POP where the vagina and cervix are descended, stretched, and herniated through the vaginal canal^10^. This animal model can serve as a useful tool to evaluate vaginal connective tissue remodeling associated with POP progression, and to determine how changes in elastic fiber content with altered fibulin-5 expression (i.e., deficiency and haploinsufficiency) affects vaginal function. Vaginal wall integrity in fibulin-5 deficient mice with and without POP was previously investigated with uniaxial biomechanical tests^28^. However, in vivo the vagina is loaded multiaxially due to pressure within the body and interactions with surrounding organs. Vaginal wall material stiffness^18,29–31^ and contractility^18,29^ are different for the circumferential versus axial directions; hence, biaxial biomechanical tests are needed to recapitulate multiaxial loading and simultaneously quantify biomechanical properties in each direction. Towards this end, extension-inflation protocols, which have the ability to maintain vaginal geometry and native SMC-matrix interactions, are useful biaxial biomechanical testing methods^31–34^.

Therefore, our objective was to evaluate the effect of fibulin-5 haploinsufficiency, and deficiency with progressive POP on the biaxial contractile and passive biomechanical function of the vagina. To accomplish this biaxial extension-inflation protocols measured maximum contraction and quantified vaginal material stiffness, circumferentially and axially. Histological analysis along the circumferential and axial planes quantified elastic fiber morphology and collagen organization. Western blotting determined collagen and SMC contractile protein expression. We hypothesized that contractility and SMC contractile protein expression decreases, material stiffness increases, and elastin area fraction decreases in the fibulin-5 haploinsufficient and deficient vaginas with progressive POP as compared to wildtype controls. Elucidating the complex interactions between proteins and cells and their contribution to vaginal function is important for understanding which components contribute to the underlying processes of POP progression and may serve as potential targets for treatment and intervention.

## Methods

### Animal use and sample preparation

**Figure 1:**
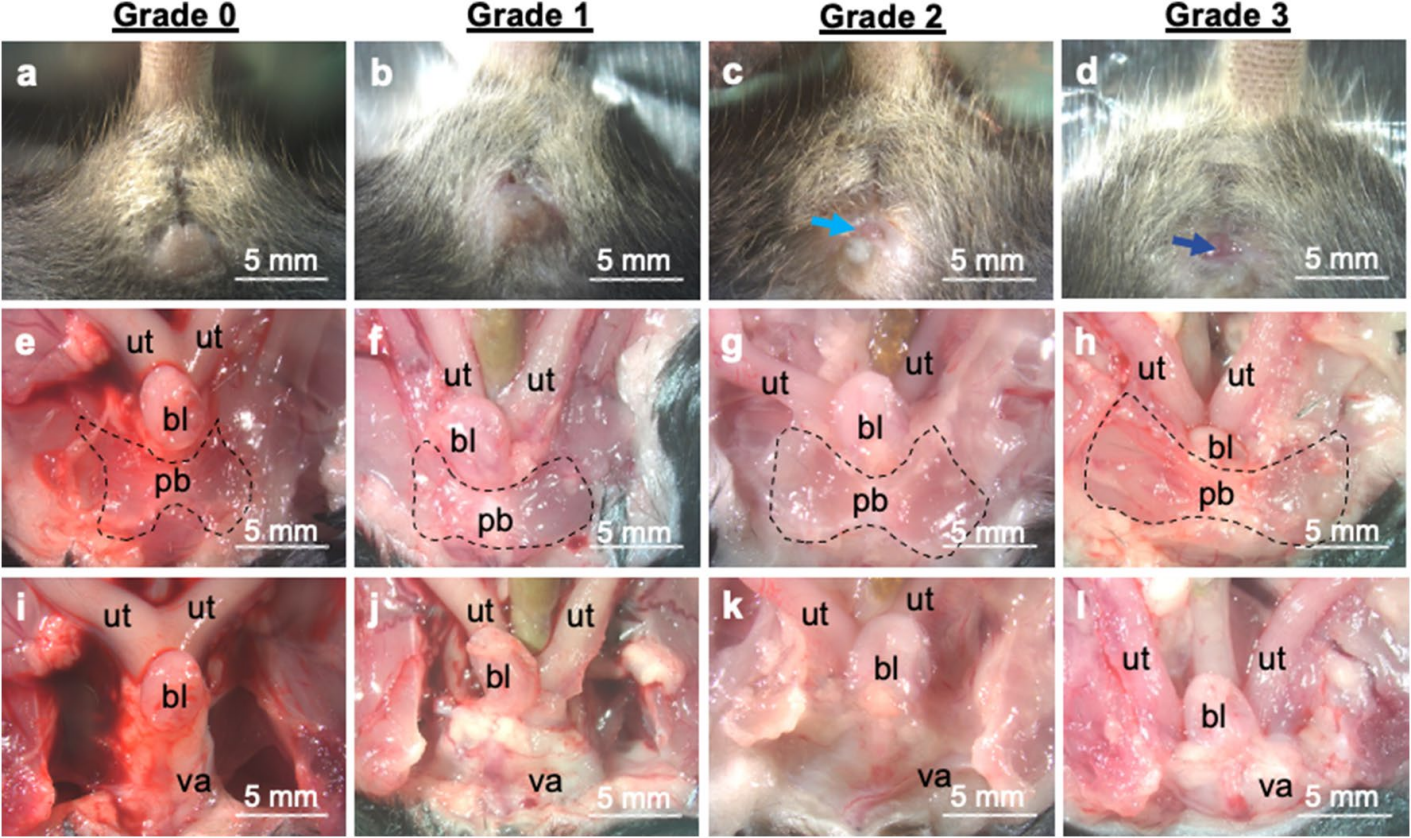
Evaluation of mouse pelvic organ prolapse. Representative images of grade 0 prolapse in the wildtype control (**a**), and grade 1 (**b**), 2 (**c**), and 3 (**d**) prolapse in the fibulin-5 deficient mice. The *Fbln5*^+/+^ and *Fbln5*^+/−^ mice did not display bulging or herniation of the pelvic organs (**a**). The bladder (bl) and bifurcation of the uterine (ut) horn were superior to the pubic bone (pb; dashed line) as shown (**e**, **i**). Mice with grade 1 prolapse displayed a small but detectable perineal bulge (**b**). Mice with grade 2 prolapse displayed bulging and herniation (light blue arrow) of the pelvic organs (**c**). In mice with grade 1 and 2 prolapse the bladder was superior to the pubic bone (**f**, **g**, **j**, **k**). In mice with grade 1 and 2 prolapse bifurcation of the uterine horn was inferior to the pubic bone descended towards the vagina (va). In grade 3 prolapse (**d**) the cervix was visualized at the vaginal opening (dark blue arrow) with downward descent of the bladder (**h**, **l**).

All procedures in this study received approval by the Institute Animal Care and Use Committee at Tulane University. This study performed all procedures in accordance with the relevant guidelines and regulations. Further, this study was conducted in compliance with the ARRIVE guidelines. The fibulin-5 (*Fbln5)* global knockout mice were developed by Dr. Hiromi Yanagisawa^35^. The *Fbln5* colony was established at Tulane University from male and female breeders supplied by Dr. Jay Humphrey from Yale University. Female and male *Fbln5* haploinsufficient mice on mixed background (C57BL/6 × 129SvEv) generated all female wildtype (*Fbln5*^+*/*+^), haploinsufficient (*Fbln5*^+/−^), and deficient (*Fbln5*^−/−^) mice used within this study (Supplementary Fig. S1). Microisolators housed all mice with littermates (no more than 5 per cage) under standard conditions with 12-h light and dark cycles. All mice were genotyped by tail snips taken during weaning by a commercial vendor (Transnetyx, Cordova, TN, USA) using real time PCR. Motivated by the observation that mechanical function correlates with the severity of POP in humans^23^, the established Mouse Pelvic Organ Prolapse Quantification (MOPQ) System^36^ divided the *Fbln5*^−/−^ mice into two groups based on POP grade: grade 1 versus grade 2 and 3 POP (Fig. 1)^37,38^. The grade 2 (n = 5) and 3 (n = 4) POP groups were combined due to statistical analysis indicating no significant differences in contractility and mechanical properties between the two groups. This minimized the number of mice needed according to ethical guidelines^39^. Further, previous work in the fibulin-5 mouse model combined grades 2 and 3 POP into a single group thus making this study comparable to the previous studies^37,38^. The individual animal served as the experimental unit throughout this study. None of the mice in this study presented with rectal prolapse.

A guillotine euthanized 44 mice without anesthesia at 3–6 months of age at estrus (by visual assessment) to enable consistent and repeatable assessment of SMC contractility and biomechanical testing (*Fbln5*^+*/*+^ n = 13, *Fbln5*^+/−^ n = 13, *Fbln5*^−/−^ with grade 1 POP n = 9 and *Fbln5*^−/−^ with grade 2/3 POP n = 9). A power analysis (*Fbln5*^+*/*+^ n = 3, *Fbln5*^+/−^ n = 3, *Fbln5*^−/−^ with grade 1 POP n = 3 and *Fbln5*^−/−^ with grade 2/3 POP n = 3; α = 0.05, β = 0.80) demonstrated that this study needed at least 7 mice per genotype and POP grade to detect significance in contractility. The total number of mice per genotype and POP grade exceeded 7 due additional preliminary studies (enzymatic digestion and gene expression not reported herein) performed after biomechanical testing. Visual assessment presented difficulties in assessing estrous cycle in the grade 2 and 3 POP mice due to the descent of the pelvic organ to the level of the introitus. The 3–6 months age range was selected due to the *Fbln5* mice developing POP as early as 3 months of age and 92% by 6 months^10^. In this study, all fibulin-5 deficient mice developed at least grade 1 POP by 3 months of age. Further, 3–6 months corresponds to 20—30 human years^40^. This is past development but not affected by senescence, therefore, representing the sexually mature adult group. In addition, statistical analysis in the *Fbln5*^+/+^ controls demonstrated that age did not significantly affect contractility and mechanical properties between 3–4 (n = 6) versus 5–6 (n = 7) months of age. Following mechanical testing 12 samples were fixed for histological analysis (n = 3/group). Carbon dioxide inhalation euthanized a separate cohort of 24 mice for western blotting and liquid nitrogen snap froze the samples (n = 6/group). Then the samples were stored at – 80 °C until western blotting analysis. A power analysis (n = 3/group, α = 0.05, β = 0.80) demonstrated that this study needed at least 6 mice per group to detect significance in protein expression. This study euthanized a total of 68 animals using littermate controls when possible. The animal distributions by genotype and prolapse grade are reported with the animal weight as mean ± standard error of mean: 19 *Fbln5*^+*/*+^ (24.1 ± 0.4 g), 19 *Fbln5*^+/−^ (23.7 ± 0.3 g), 15 *Fbln5*^−/−^ with grade 1 POP (23.7 ± 0.6 g), 8 *Fbln5*^−/−^ with grade 2 POP (24.5 ± 0.2 g), and 7 *Fbln5*^−/−^ with grade 3 POP (24.1 ± 0.3 g).

### Balloon catheterization

At least one estrous cycle (at estrus) prior to experimentation a balloon catheter measured intravaginal pressures in 3–6 months of age female *Fbln5*^+*/*+^ (n = 6), *Fbln5*^+/−^ (n = 6), and *Fbln5*^−/−^ with grade 1 POP (n = 5) mice^18,41,42^. A custom polyvinyl chloride 3 mm-diameter balloon was attached to a 1.25 mm-diameter aluminum tube to construct the balloon catheter^18^. The balloon catheter was then connected to a 3-way stop cock, 3-mL syringe, and pressure transducer (-50 to + 300 mmHg; product number MLT0699; ADInstruments, Colorado Springs, CO). The pressure transducer connected to a laptop and system components (PowerLab and LabChart8, ADInstruments, Colorado Springs, CO, USA). The 3-mL syringe filled the aluminum tube with water to distend the balloon to 3 mm and obtain a baseline pressure reading. The balloon inserted into the vaginal canal by passing the introitus of the anesthetized mice (4% isoflurane in 100% oxygen). The pressure transducer measured the current pressure and the change between the current and baseline pressure quantified the in vivo pressure^18,43^. All animals underwent balloon catheterization 3 times to average intravaginal pressure readings minimizing user variability^18^. The *Fbln5*^−/−^ mice with grade 2 or 3 POP did not undergo balloon catheterization due to cervical descent through the vaginal canal to the level of the introitus (Fig. 1). A separate cohort of *Fbln5*^−/−^ mice with grade 1 POP underwent biomechanical testing due to POP progression readily increasing to stage 2 or 3 POP within 3 days and microstructural analysis suggesting that mild distension of that the *Fbln5*^−/−^ vagina with a balloon catheter induced collagen remodeling (Supplemental Fig. S2)^44^. Contractility and mechanical properties were not significantly different in the *Fbln5*^+/+^ and *Fbln5*^+/−^ mice between the balloon catheterized mice (n = 6) and controls (n = 7). Balloon catheterization permitted measuring the in vivo vaginal pressures (*Fbln5*^+*/*+^ = 7 ± 2 mmHg, *Fbln5*^+/−^ = 5 ± 1 mmHg, and *Fbln5*^−/−^grade 1 POP = 9 ± 2 mmHg; mean ± standard deviation) to recapitulate the loads in vitro during testing. The mean vaginal pressure from grade 1 POP mice was used for grade 2 and 3 POP during contractile and mechanical assessment.

### Maximum contractility and biaxial biomechanical testing

**Figure 2:**
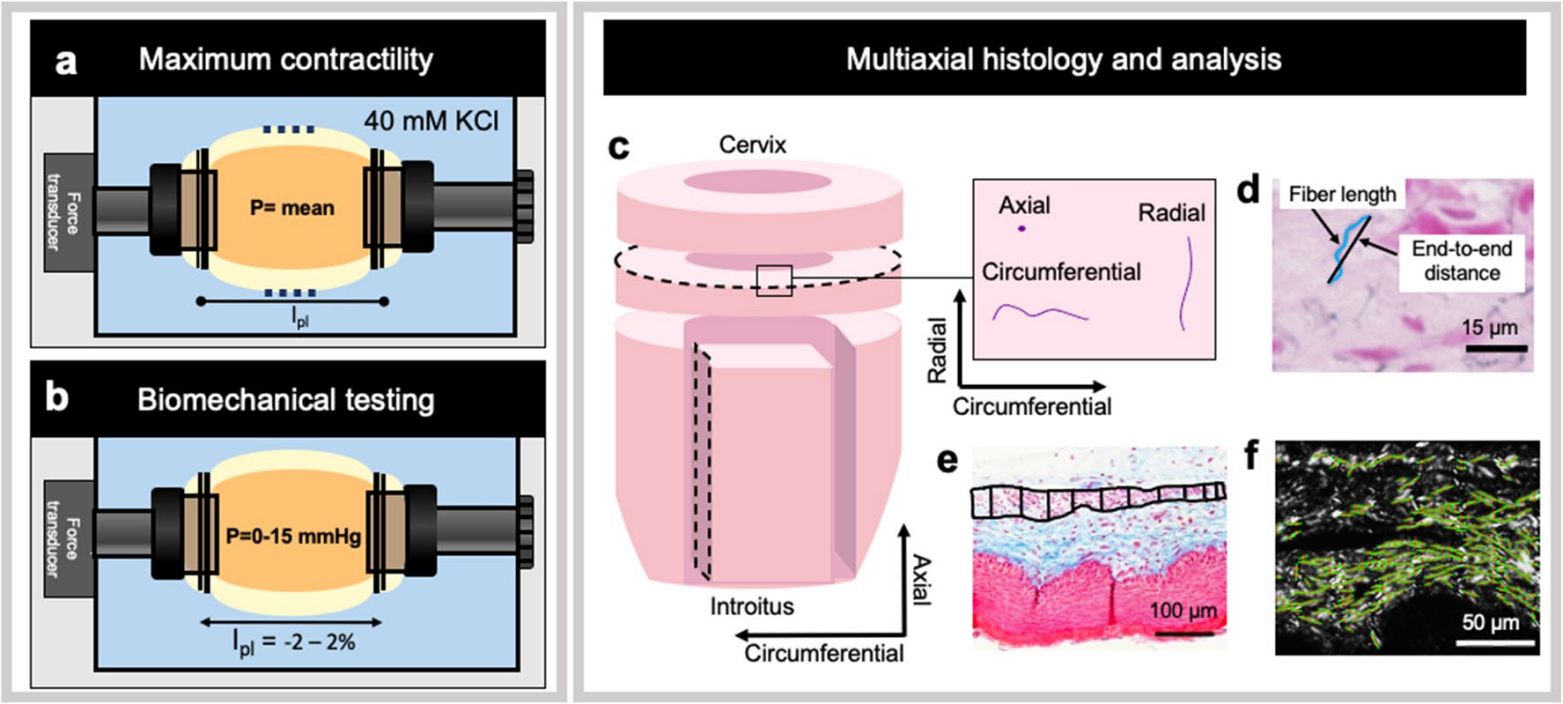
Schematic of experimental design. Schematic of extension-inflation device (**a**, **b**). A video-microscope (dashed lines) optically tracked the outer diameter and a force transducer measured axial force. At the physiologic length (l_pl_) and mean in vivo pressure (P) 40 mM of potassium chloride (KCl) induced maximum SMC contraction (**a**). For passive biaxial biomechanical testing, the vagina was subjected to increasing vaginal pressures at various fixed axial lengths (**c**). Circumferential and axial sections randomly taken from the proximal or distal region of the vagina (not depicted) permitted multiaxial histological analysis (**c**). When analyzing the circumferential plane fibers oriented towards the circumferential and radial directions. Fibers in the axial direction were perpendicular to the circumferential plane and appeared as small dots (**d**). Representative zoomed in Hart's elastin image measuring the fiber length and end-to-end distance to quantify elastic fiber tortuosity (**d**). ImageJ quantified muscularis thickness using the Masson’s trichrome stained images (**e**). Representative zoomed in Picrosirius red image showing the extracted fiber features using the CurveAlign open-source software for bulk assessment to quantify collagen fiber alignment (**f**).

Biaxial extension-inflation protocols quantified the biaxial contractile (Fig. 2a) and biomechanical properties (Fig. 2b) under physiologically relevant loads in the *Fbln5*^+*/*+^ (n = 13), *Fbln5*^+/−^ (n = 13), *Fbln5*^−/−^ with grade 1 POP (n = 9) and *Fbln5*^−/−^ with grade 2/3 POP (n = 9) vaginas^12,17,18,31–34,45^. This method permitted maintenance of intact organ geometry and native smooth muscle cell-extracellular matrix (SMC-ECM) interactions. Immediately following sacrifice, the vagina was dissected with forceps removing the urethra and paravaginal fascia. Scissors then separated the vagina from the cervovaginal complex in 4 °C Hank’s Balanced Salt Solution^31^. Two 6–0 sutures mounted the *Fbln5*^+*/*+^, *Fbln5*^+/−^, and *Fbln5*^−/−^ with grade 1 POP vaginas onto 3.75 mm-diameter cannulas in the pressure-myograph system (Danish Myo Technology, Aarhus, Denmark). Due to the vaginas from the *Fbln5*^−/−^ with grade 2 or 3 POP being larger in diameter 5.00 mm-diameter cannulas were used for this group. The vagina was submerged in Krebs Ringer Buffer (KRB; 120 mM NaCl, 25 mM NaHCO_3_, 4.7 mM KCl, 2.5 mM CaCl_2_, 1.2 mM NaH_2_PO_4_, 1.2 mM MgCl_2_, 11 mM glucose) at 37 °C aerated with 95% ${\text{O}}_{2}$ and 5% ${\text{CO}}_{2}$ to maintain pH at 7.4^17,46^.

Identification of the vaginal unloaded length and outer diameter occurred where the organ slightly buckled and did not collapse, respectively, as illustrated previously by Robison et al.^31^. Explanting the vagina and removing the natural tethering, such as the paravaginal fascia, resulted in the vagina retracting (i.e., shortening in length). Therefore, the linear stage axially extended the vagina to the physiologic length^34^. Measuring the in vivo to ex vivo change in vaginal axial length between stain lines placed during dissection initially estimated retraction of the vagina^31,34,45^. Upon identifying the unloaded configuration within the device, a digital caliper (Mitutoyo, #CD-6”-ASX, Kawasaki, Kanagawa, Japan) measured the suture to suture length. The force transducer further evaluated the physiologic length wherein the transducer-measured axial force held constant over a range of increasing pressure from 0 to 15 mmHg preserving energy as previously described^31,33,47^. An integrated system of components (Eclipse TS100 Microscope, Nikon, Melville, NY, USA) and software (Myoview Software, Danish Myo Technology, Aarhus, Denmark) recorded the outer diameter and transducer-measured axial force during mechanical testing. The linear micrometer returned the vagina to its unloaded length to pre-expose the smooth muscle cells to potassium-chloride (KCl) for contraction.

The SMCs were first preconditioned under low loads about the in vivo pressure to ensure viability. Intraluminal pressure was set to one standard deviation below the mean measured in vivo pressure (*Fbln5*^+*/*+^ = 5 mmHg, *Fbln5*^+/−^ = 4 mmHg, and *Fbln5*^−/−^grade 1 and 2/3 POP = 7 mmHg)^17,18,48^. The linear stage axially extended the vagina from the unloaded length to the length wherein the transducer-measured force equalled zero. The SMCs were stimulated with 40 mM of KCl for 5 min to achieve a steady maximum contractile response^18,49^. Fresh KRB washed the vagina and returned SMC tone to the basal state. KCl induces muscle contraction through direct membrane depolarization of the SMC by opening voltage dependent calcium channels resulting in an influx of extracellular calcium. This simple mechanism of contraction served as initial interest because it induces a tonic contractile response and is independent of neural function and the number or receptor available^18,49,50^. This was then repeated at an intraluminal pressure one standard deviation above the mean measured in vivo pressure (*Fbln5*^+*/*+^ = 9 mmHg, *Fbln5*^+/−^ = 6 mmHg, and *Fbln5*^−/−^grade 1 and 2/3 POP = 11 mmHg). Five cycles of pressurization (0–15 mmHg; 1.5 mmHg) at the physiologic length preconditioned the vagina to obtain a consistent and repeatable biomechanical response. Five cycles of axial extension (10 µm/s) from 2% below to 2% above the physiologic length at 5 mmHg (1/3 maximum pressure) further preconditioned the vagina^12,18,31,34^. The vagina equilibrated at the physiologic length and 5 mmHg (1/3 maximum pressure) for 10 mins^31^. The linear stage re-established the unloaded length and the video-camera recorded the outer diameter^18,31^.

At the physiologic length and in vivo measured mean pressure, 40 mM of KCl maximally contracted the vagina (Fig. 2a)^17,18,46^. The video-microscope and force transducer measured the outer diameter and axial force, respectively, at the basal state and when maximally contracted. After maximum contraction, fresh KRB returned SMC tone to the basal state. The vagina underwent pressure-diameter tests with 5 cycles of pressurization (0–15 mmHg; 1.5 mmHg/s) at the physiologic length and 2% above and below the physiologic length (Fig. 2b)^18,31,34^. Then the vagina underwent force–length tests with 3 cycles of axial extension from 2% below to 2% above the physiologic length under 4 constant pressures: a tare load (P = 2 mmHg), 1/3 maximum pressure (P = 5 mmHg), 2/3 maximum pressure (P = 10 mmHg), and maximum pressure (P = 15 mmHg)^31,33,34^. Intersection of the force–length response for most of the pressures confirmed the physiologic length^31,33,47^. The vagina incubated for 30 min in calcium-free KRB with a calcium-chelating agent (2 mM egtazic acid; EGTA) to eliminate SMC tone for passive biomechanical testing^18,51^. After eliminating SMC tone the vagina underwent the same biaxial biomechanical testing protocols under the passive condition. In this study the passive (i.e., collagen and elastic fiber contribution) mechanical properties were of primary interest.

### Mechanical testing data analysis

During mechanical testing an 40 MHz ultrasound transducer (Vevo2100; LZ550 center frequency transducer; FUJIFILM VisualSonics, Inc., Toronto, Canada) in short-axis B-mode measured the unloaded thickness at the middle-third vagina^18,34^. The video microscope unloaded outer radius (${R}_{o}$), ultrasound unloaded thickness ($H$), and suture to suture unloaded axial length (L) calculated the volume [$\overline{V }$; Eq. (1)]. Under the assumption that volume remains constant throughout the test, the deformed inner radius [${r}_{i}$; Eq. (2)] and thickness [$h$; Eq. (3)] were calculated throughout the experiment^18,34^.

1 $$\overline{V} = \pi \left( {R_{o}^{2} - \left( {R_{o} - H} \right)^{2} } \right)L$$

2 $$r_{i} = \sqrt {r_{o}^{2} - \frac{{\overline{V}}}{\pi l}}$$

3 $$h = r_{o} - r_{i}$$

The intraluminal pressure ($$P)$$, transducer-measured axial force ($${F}_{t}$$), inner radius, and outer radius calculated the wall-averaged Cauchy stress ($$\sigma$$) for the circumferential [$$\theta$$; Eq. (4)], and axial [z; Eq. (5)] directions^33,52^.

4 $$\sigma_{\theta } = \frac{{Pr_{i} }}{{r_{o} - r_{i} }}$$

5 $$\sigma_{z} = \frac{{F_{t} + \pi Pr_{i}^{2} }}{{\pi \left( {r_{o}^{2} - r_{i}^{2} } \right)}}$$

The radius at the mid-wall of the deformed state to the mid-wall radius at the physiologic length under 0 mmHg defined the circumferential stretch ratio [${\lambda }_{\theta }$; Eq. (6)]^33^. The deformed axial length with respect to the unloaded axial length calculated the axial stretch ratio [${\lambda }_{z}$; Eq. (7)].

6 $$\lambda_{\theta } = \frac{{r_{i} + h/2}}{{R_{i} + H/2}}$$

7 $$\lambda_{z} = \frac{l}{L}$$

The passive stress versus stretch behavior is reported at the physiologic length. The slope of a linear line fitted to the stress versus stretch curves between 1 standard deviation above and below the in vivo pressure range quantified material stiffness (Supplementary Fig. S3)^18,34,53^. To compare contractility independent of geometry the change in stress was evaluated. The circumferential stress when maximally contracted was subtracted from the circumferential stress at the basal state ($\Delta$ circumferential stress) to quantify circumferential contractility. The axial stress when maximally contracted was subtracted from the axial stress at the basal state ($\Delta$ axial stress) to quantify axial contractility^17,18^.

### Multiaxial histological analysis

After the mechanical test, 10% formalin perfusion fixed the *Fbln5*^+*/*+^ (n = 3), *Fbln5*^+/−^ (n = 3), *Fbln5*^−/−^ with grade 1 POP (n = 3), and *Fbln5*^−/−^ with grade 2/3 POP (n = 3) vaginas at the physiologic length and mean in vivo pressure for 24 h, followed by embedding the samples in paraffin. Circumferential and axial sections of 4 µm thickness were randomly taken from the proximal or distal region of the vagina and stained with Masson’s Trichrome (MTC), Picrosirius Red (PSR), and Hart’s Elastin stains to evaluate the microstructure (Fig. 2c)^18^. An Olympus BX51 Microscope, an Olympus DP27 Digital Camera, and cellSens Software (Olympus Corporation, Center Valley, PA, USA) took brightfield (MTC and Harts) and polarized darkfield (PSR) images. Hart’s Elastin stain was imaged under 40 × magnification at 4 random locations along the section. In ImageJ the total elastic fiber length was measured within the muscularis and subepithelial layer (U.S. National Institutes of Health, Bethesda, ME, USA) of the vaginal wall using the segmented line tool. The straight-line tool in ImageJ manually measured the end-to-end fiber distance (Fig. 2d). Elastic fiber tortuosity was quantified for each fiber by dividing the total fiber length to the end-to-end distance^54,55^. This described the straightness of the elastic fiber with a value of 1 denoting a straight fiber (Fig. 2d). Across all four images (425 × 333 µm) for each sample on average a total of 50–100 fibers (per plane and layer) were analyzed for the *Fbln5*^+/+^ and *Fbln5*^+/−^ vaginas, and 2–13 fibers for the *Fbln5*^-/-^ vaginas. A custom protocol using GNU Image Manipulation Program (GIMP) quantified elastin area fraction independently within the subepithelium and muscularis^12,56^. The subepithelium was outlined to determine the total number of pixels within this area. In GIMP the subepithelium was isolated and the color selection tool was used to select a representative elastin pixel (dark purple) then all pixels of similar values were automatically selected. The number of elastin pixels in the subepithelium divided by the total number of pixels in the subepithelium quantified elastin area fraction for the subepithelial layer. This was repeated for the muscular layer. MTC stain was imaged under 40 × magnification at four random locations along the section. Vaginal muscularis thickness was manually calculated on each section using ImageJ by identifying the muscular layer and drawing 10 lines across it (Fig. 2e)^57,58^. This was performed on all four 40 × images then averaged for each sample. PSR stain was imaged under 40 × magnification at four random locations along the section. The open source CurveAlign software (Laboratory for Optical and Computational Instrumentation, Madison, WI, USA; http://www.loci.wisc.edu/software/curvealign) quantified the collagen fiber alignment ratio with a value of 1 denoting highly aligned fibers towards a preferred direction (Fig. 2f)^59,60^. This was performed on all four 40 × images then averaged for each sample. The multiaxial histology approach permitted evaluating the circumferential and radial (across the thickness) oriented fibers along the circumferential section. The axial fibers were perpendicular to the circumferential plane appearing as small dots and vice versa for the axial section. This method does not permit simultaneously evaluating the circumferential and axial oriented fibers within the same plane. Further the radial fibers were included in the analysis.

### Western Blot analysis

Western blots were performed as described previously^61–63^. Briefly, lysates were prepared from *Fbln5*^+*/*+^ (n = 6), *Fbln5*^+/−^ (n = 6), *Fbln5*^−/−^ with grade 1 POP (n = 6) and *Fbln5*^−/−^ with grade 2/3 POP (n = 6) vaginal tissues by manual homogenization and sonication in Mammalian Cell Lysis Buffer (Abcam, Cat No. ab179835) with Halt Protease and Phosphatase Inhibitor Cocktail (100X) (Thermo Fisher Scientific, Cat No. PI78440). Protein concentration was measured using Bradford assay and 20 μg of protein for each lysate was prepared with sample loading buffer (4 × Laemmli Sample Buffer, Bio-Rad, Cat No. 1610747) containing β-mercaptoethanol. Samples were separated on 4–20% sodium dodecyl sulphate–polyacrylamide gel (Mini-PROTEAN TGX, Bio-Rad, Cat No. 456–1093) and transferred to PVDF membrane (Bio-Rad, Cat No. 1620177). Based on the antibody used, membranes were blocked with either 5% milk or 5% BSA in TBST (100 mm NaCl, 0.1% Tween 20, 50 mm Tris, pH 7.4) for 1 h at room temperature followed by overnight incubation with primary antibody at 4 °C. The primary antibodies used were rabbit anti-Collagen I antibody (Abcam, Cat No. ab34710), rabbit anti-Collagen III (Abcam, Cat No. ab7778), rabbit anti-Myosin smooth muscle heavy chain 1 and 2 (Abcam, Cat No. ab124679), rabbit anti-Alpha smooth muscle actin (Abcam, Cat No. ab5694), rabbit anti-GAPDH (Cell Signal Technology, Cat No. 5174S) in 1:1000 dilution. Thereafter, membranes were washed with TBST and incubated with (1:5000) horseradish peroxidase-conjugated goat anti-rabbit or anti-mouse antibody (Bio-Rad) for 1 h at room temperature. Protein detection was done using ECL-Plus kit (GE Healthcare, Amersham, Cat No. 45002401) and iBright gel imager (Thermo Fisher Scientific). Immunoblot data were quantified by computer-aided densitometry analysis using ImageJ software, data normalized to GAPDH levels and expressed as relative increase *vs Fbln5*^+*/*+^, as described previously^62,63^.

### Statistical analysis

Prior to performing statistical analysis, the data was checked for normality with 3 assumptions: (1) homogeneity of variances by Levene’s test, (2) test of normality by Shapiro–Wilk test, and (3) detection of outliers. Using a box and whisker plot, values greater than 3 times the interquartile range were considered outliers^64^. These criteria were established a priori. For small sample size [i.e., histology (n = 3) and western blots (n = 6)] a Dixon’s Q test evaluated outliers. No individual animal was excluded from the analysis. When the normality assumption was satisfied, a One-way ANOVA evaluated the effect of fibulin-5 insufficiency and POP on contractility, passive mechanical properties, histological microstructure, and protein expression, followed by Tukey’s post-hoc test. For non-normally distributed data, a nonparametric Kruskal–Wallis test was used, followed by post-hoc Dunn’s test. Motivated by previous work demonstrating a correlation between mechanical properties and prolapse stage in humans the *Fbln5* mice were divided by POP grade^23^. The *Fbln5*^+/+^ and *Fbln5*^+/−^ mice were designated as grade 0 POP. Pearson’s or nonparametric Spearman’s correlations evaluated the relationship between contractility and passive mechanical properties with respect to prolapse grade. Motivated by prior work within other tissues demonstrating that contractility correlated with the passive mechanical properties, Pearson’s and nonparametric Spearman’s test evaluated the correlation between passive mechanical properties and contractility^65^. Motivated by prior work suggesting that elastic fibers may contribute to SMC contractility^15–18^, Pearson’s or Spearman’s test evaluated the correlation between contractility and elastin area fraction. R Statistical Software performed statistical analyses. Level for statistical significance was set to p < 0.05. Results are reported as mean ± standard error of the mean (SEM) or box and whisker plots.

## Results

### Vaginal contractility with fibulin-5 insufficiency and POP

**Figure 3:**
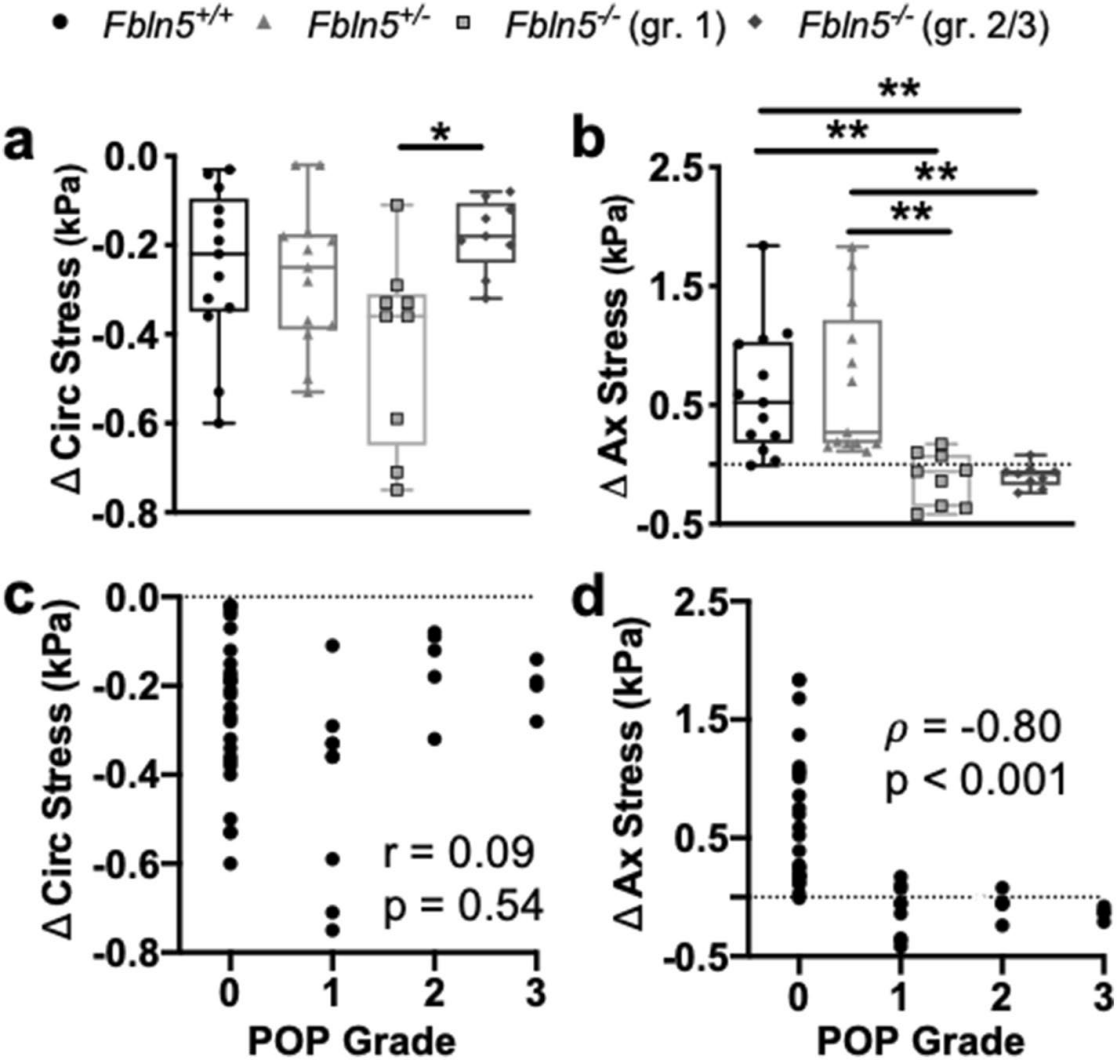
Changes in vaginal wall stress with maximum contractility. Changes in circumferential stress (**a**; circ) quantifying maximum circumferential contractility reported as box and whisker plots. The box and whisker plots graphically display the median, lower and upper quartiles, and lower and upper extremes of data in the *Fbln5*^+/+^ (circle; n = 13), *Fbln5*^+/−^ (triangle; n = 13), *Fbln5*^-/-^ with grade 1 POP (square; n = 9), and *Fbln5*^-/-^ with grade 2/3 POP (diamond; n = 9). Fibulin-5 insufficiency and POP affected changes in circumferential stress (p = 0.02). Changes in circumferential stress decreased in the *Fbln5*^−/−^ with grade 2/3 POP compared to grade 1 POP (p = 0.02). Changes in axial stress (**b**; ax) quantifying maximum axial contractility. Fibulin-5 insufficiency and POP affected changes in axial stress (p < 0.001). Changes in axial stress decreased in the *Fbln5*^−/−^ with grade 2/3 POP compared to the *Fbln5*^+*/*+^ (p = 0.005) and *Fbln5*^+/−^ (p = 0.003) vaginas. Changes in axial stress decreased in the *Fbln5*^−/−^ with grade 1 POP compared to the *Fbln5*^+*/*+^ (p = 0.009) and *Fbln5*^+/−^ (p = 0.005) vaginas. In addition to identifying significant differences in change in stress, the data was separated according to POP grade: 0 (n = 26), 1 (n = 9), 2 (n = 5), 3 (n = 4). Pearson’s and nonparametric Spearman’s test evaluated the correlation between the changes in stress with POP grade. Changes in circumferential (**c**) and axial (**d**) stresses reported as a function of POP grade. Changes in axial stress significantly negatively correlated with POP grade. Statistical significance is denoted by *p < 0.05 and **p < 0.01.

A one-way ANOVA showed that fibulin-5 insufficiency and POP affected changes in circumferential stress (p = 0.02 Fig. 3a). Tukey’s post-hoc test demonstrated that changes in circumferential stress decreased (p = 0.02) in the *Fbln5*^−/−^ with grade 2/3 compared to the grade 1 POP. A nonparametric Kruskal–Wallis test revealed that fibulin-5 insufficiency and POP affected changes in axial stress (p < 0.001; Fig. 3b). Dunn’s post-hoc test demonstrated that changes in axial stress decreased in the *Fbln5*^−/−^ with grade 2/3 POP compared to the *Fbln5*^+*/*+^ (p = 0.005) and *Fbln5*^+/−^ (p = 0.003) vaginas. Further, that changes in axial stress decreased in the *Fbln5*^−/−^ with grade 1 POP compared to the *Fbln5*^+*/*+^ (p = 0.009) and *Fbln5*^+/−^ (p = 0.005) vaginas. Other comparisons were not statistically significant. In addition to identifying significant differences the data was split based on POP grade to determine the relationship between contractility and POP grade. The Pearson correlation did not identify a significant relationship between changes in circumferential stress and POP grade (p = 0.54, r = 0.09; Fig. 3c). Changes in axial stress negatively correlated with POP grade (p < 0.001, ρ = − 0.80; Fig. 3d), therefore, as POP grade increased axial contractility decreased.

### Vaginal passive biomechanical function with fibulin-5 insufficiency and POP

**Figure 4:**
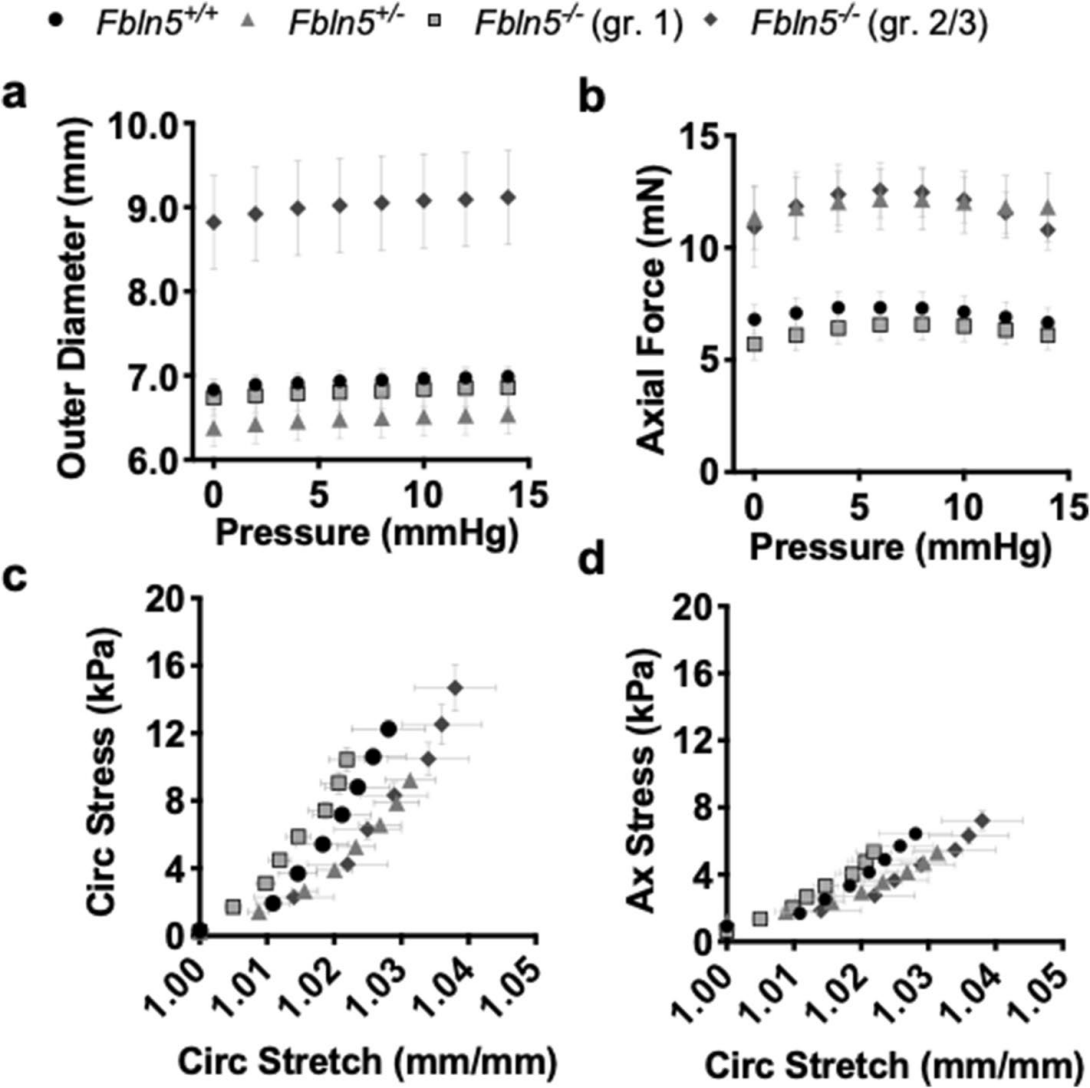
Curves for vaginal passive biomechanical behavior. Pressure versus outer diameter curves (**a**) for the *Fbln5*^+/+^ (circle; n = 13), *Fbln5*^+/−^ (triangle; n = 13), *Fbln5*^-/-^ with grade 1 POP (square; n = 9), and *Fbln5*^-/-^ with grade 2/3 POP (diamond; n = 9) vaginas. The *Fbln5*^-/-^ with grade 2/3 POP vaginal diameter increased compared to the other groups denoted by the upward shift in the pressure versus diameter curve. Pressure versus axial force curve (**b**) for the *Fbln5*^+/−^ and *Fbln5*^-/-^ with grade 2/3 POP shifted upwards compared to *Fbln5*^+/+^ and *Fbln5*^-/-^ with grade 1 POP. Circumferential (**c**; circ) and axial (**d**; ax) stress versus circumferential stretch curves. Vaginal distensiblility increased for *Fbln5*^+/−^ and *Fbln5*^-/-^ with grade 2/3 POP with respect to *Fbln5*^+/+^ and *Fbln5*^-/-^ with grade 1 POP as denoted by the rightward shift in the stress versus stretch curves. Data is reported as mean ± standard error of mean. Less points are plotted than used in the analysis and the error bars are a light grey in order to visualize the data.

The pressure-outer diameter curve demonstrated dilation of the *Fbln5*^-/-^ vaginal outer diameter with grade 2/3 POP compared to all other groups as denoted by the upward shift in the curve (Fig. 4a). A one-way ANOVA revealed that fibulin-5 insufficiency and POP significantly affected (p < 0.001) the unloaded and physiologic outer diameter at the mean in vivo pressure and physiologic length (Supplementary Table S1). Tukey’s post-hoc test showed that the *Fbln5*^−/−^ vagina with grade 2/3 POP presented a larger physiologic outer diameter (p < 0.001) than the *Fbln5*^+*/*+^, *Fbln5*^+/−^, and *Fbln5*^−/−^ with grade 1 POP. In addition to identifying significant differences the data was divided based on POP grade to determine the relationship between geometry and POP grade. A Pearson’s correlation demonstrated that the physiologic outer diameter positively correlated with POP grade (r = 0.68, p < 0.001; Supplementary Fig. S4), therefore, as POP grade increased the outer diameter increased. The *Fbln5*^+/−^ and *Fbln5*^−/−^ with grade 2/3 POP pressure versus axial force curve shifted upwards compared to the *Fbln5*^+*/*+^ and *Fbln5*^−/−^ with grade 1 POP (Fig. 4b). A nonparametric Kruskal–Wallis test revealed that fibulin-5 insufficiency and POP significantly (p < 0.001) affected axial force at the mean in vivo pressure and physiologic length (Supplementary Table S1). Dunn’s post hoc-test demonstrated that the *Fbln5*^−/−^ vagina with grade 2/3 POP displayed a higher axial force than the *Fbln5*^+*/*+^ (p = 0.01) and *Fbln5*^−/−^ with grade 1 POP (p < 0.001). Further, the *Fbln5*^+/−^ vagina displayed a higher axial force than the *Fbln5*^+*/*+^ (p = 0.01) and *Fbln5*^−/−^ with grade 1 POP (p < 0.001)*.* A nonparametric Kruskal–Wallis test revealed that fibulin-5 insufficiency and POP significantly affected (p = 0.004) the unloaded and physiologic vaginal length (Supplementary Table S1). Dunn’s post hoc-test demonstrated that the *Fbln5*^−/−^ vagina with grade 2/3 POP had a longer (p = 0.001) physiologic vaginal length than the *Fbln5*^−/−^ with grade 1 POP*.*

**Figure 5:**
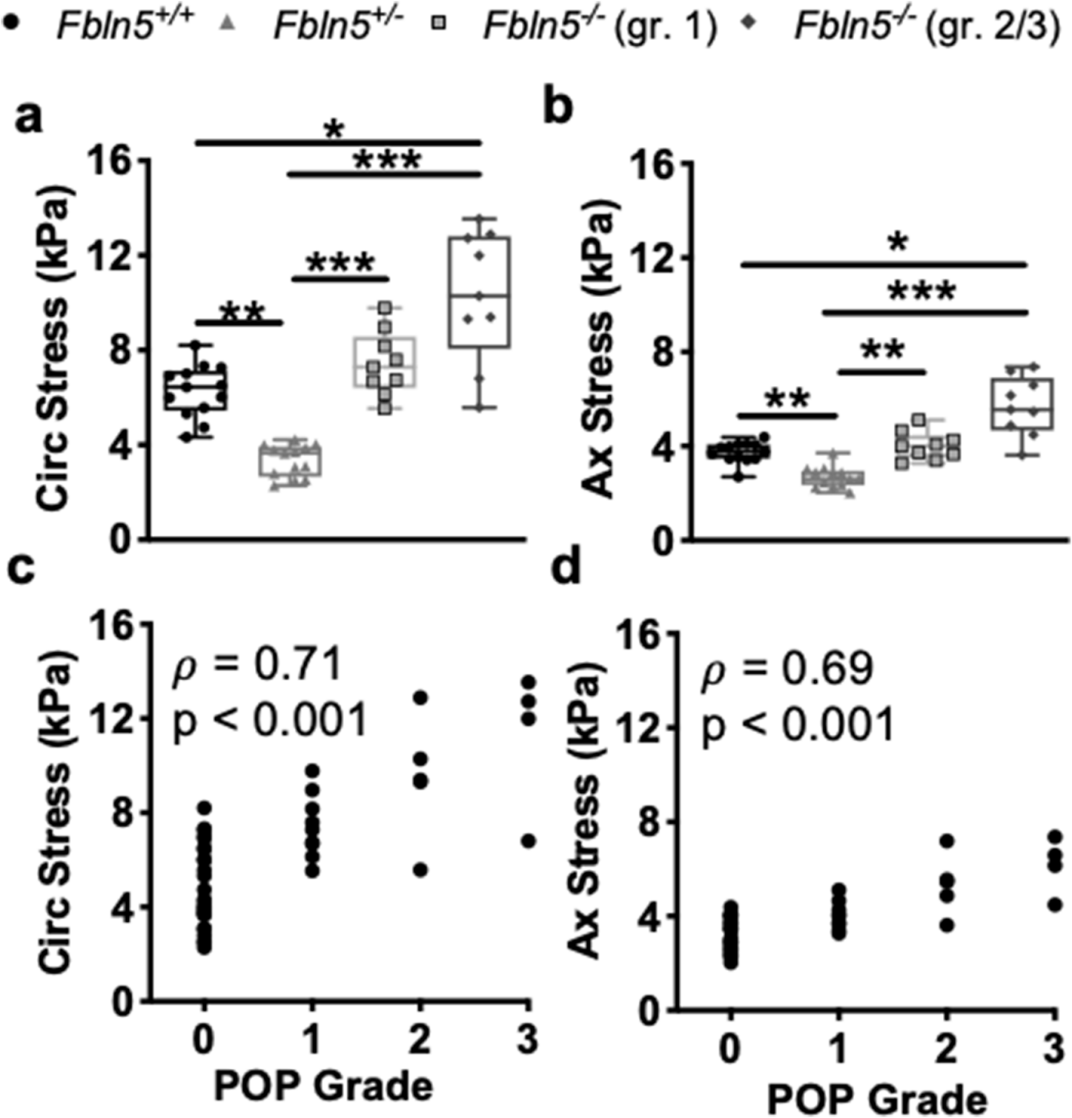
Vaginal wall physiologic stress at the mean in vivo pressures. Circumferential stress reported as box and whisker plots (**a**). The box and whisker plots graphically display the median, lower and upper quartiles, and lower and upper extremes of data in the *Fbln5*^+/+^ (circle; n = 13), *Fbln5*^+/−^ (triangle; n = 13), *Fbln5*^-/-^ with grade 1 POP (square; n = 9), and *Fbln5*^-/-^ with grade 2/3 POP (diamond; n = 9) vaginas. Fibulin-5 insufficiency and POP significantly affected (p < 0.001) circumferential stress. Circumferential stress increased in the *Fbln5*^−/−^ vagina with grade 2/3 POP compared to *Fbln5*^+*/*+^ (p = 0.04) and *Fbln5*^+/−^ (p < 0.001) vaginas*.* Circumferential stress decreased in the *Fbln5*^+/−^ vagina compared to *Fbln5*^+*/*+^ (p = 0.003) and *Fbln5*^−/−^ with grade 1 POP (p < 0.001). Axial stress reported as box and whisker plots for the *Fbln5*^+/+^, *Fbln5*^+/−^, and *Fbln5*^-/-^ with grade 1 and 2/3 POP (**b**). Fibulin-5 insufficiency and POP significantly affected (p < 0.001) axial stress. Axial stress increased in the *Fbln5*^−/−^ vagina with grade 2/3 POP compared to *Fbln5*^+*/*+^ (p = 0.03) and *Fbln5*^+/−^ (p < 0.001) vaginas*.* Axial stress decreased in the *Fbln5*^+/−^ vagina compared to *Fbln5*^+*/*+^ (p = 0.008) and *Fbln5*^−/−^ with grade 1 POP (p = 0.003). In addition to identifying significant differences in stress, the data was separated according to POP grade: 0 (n = 26), 1 (n = 9), 2 (n = 5), 3 (n = 4). Pearson’s or nonparametric Spearman’s test evaluated the correlation between stress with POP grade. Circumferential (**c**) and axial (**d**) stresses positively correlated with POP grade. Statistical significance is denoted by *p < 0.05, **p < 0.01 and ***p < 0.001.

The *Fbln5*^+/−^ and *Fbln5*^−/−^ vagina with grade 2/3 POP were more distensible than the *Fbln5*^+*/*+^ and *Fbln5*^−/−^ vagina with grade 1 POP denoted by the rightward shift in the stress-stretch curves (Fig. 4c,d). A nonparametric Kruskal–Wallis test revealed that fibulin-5 insufficiency and POP significantly affected (p < 0.001) circumferential (Fig. 5a) and axial (Fig. 5b) stresses at the physiologic length and mean in vivo pressure. Dunn’s post hoc-test showed that circumferential (p = 0.04) and axial (p = 0.03) stresses increased in the *Fbln5*^−/−^ vagina with grade 2/3 POP compared to *Fbln5*^+*/*+^*.* The circumferential and axial stresses increased (p < 0.001) in the *Fbln5*^−/−^ vagina with grade 2/3 POP compared to *Fbln5*^+/−^*.* Circumferential stress decreased in the *Fbln5*^+/−^ vagina compared to *Fbln5*^+*/*+^ (p = 0.003) and *Fbln5*^−/−^ with grade 1 POP (p < 0.001). Axial stress decreased in the *Fbln5*^+/−^ vagina compared to *Fbln5*^+*/*+^ (p = 0.008) and *Fbln5*^−/−^ with grade 1 POP (p = 0.003). In addition to identifying significant differences the data was divided based on POP grade to determine the relationship between mechanical properties and POP grade. Spearman’s correlations demonstrated that circumferential (ρ = 0.71, p < 0.001; Fig. 5c) and axial (ρ = 0.69, p < 0.001; Fig. 5d) stresses positively correlated with POP grade. Therefore, as POP grade increased vaginal wall stress increased. Further, axial stress significantly negatively correlated with changes in axial stress due to axial contractility (ρ = − 0.60, p < 0.001; Supplemental Fig. S5). Therefore, as axial stress increased axial contractility decreased. Circumferential stretch, axial stretch, circumferential material stiffness, and axial material stiffness were not statistically significant (Supplementary Table S1).

### Elastic fiber morphology and collagen organization

**Figure 6:**
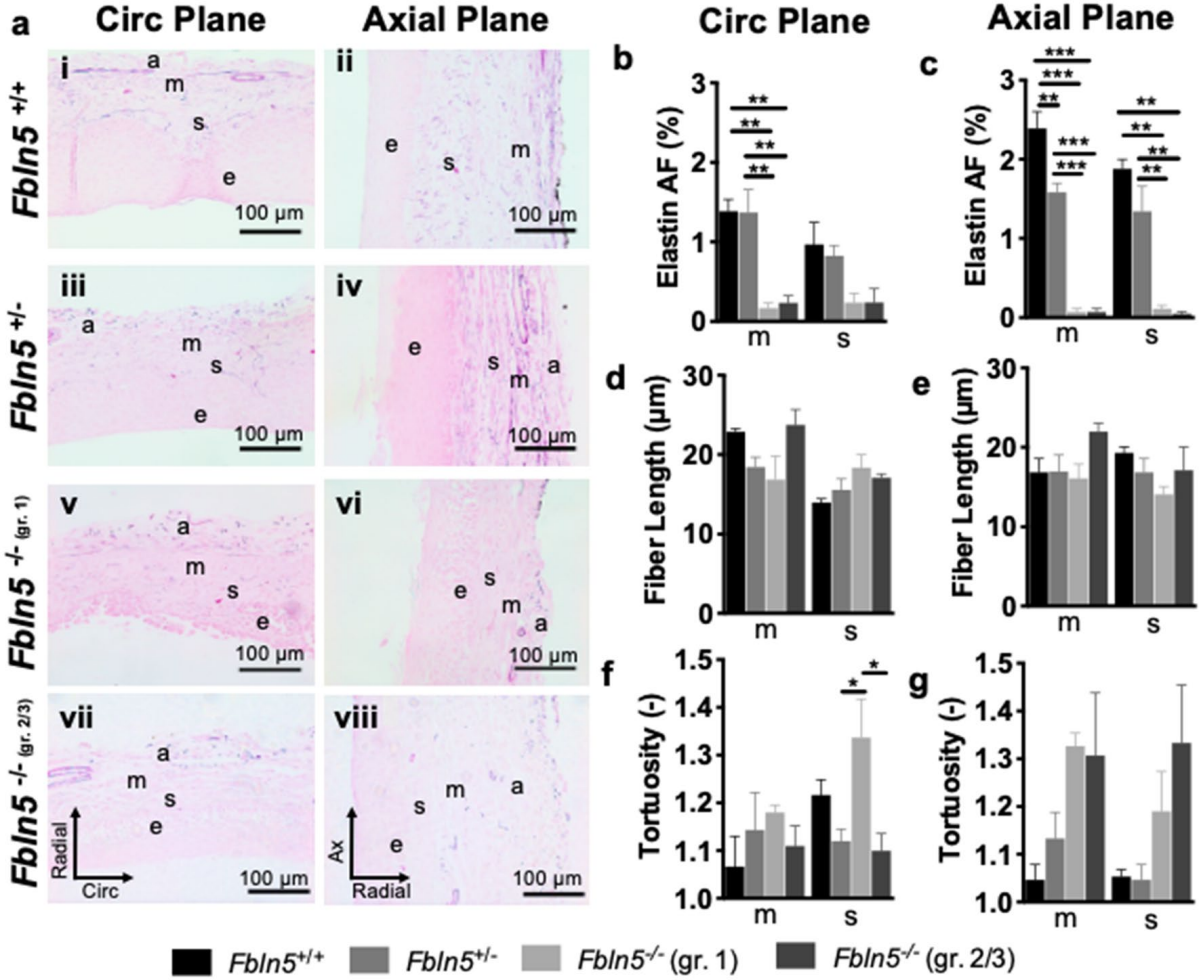
Elastic fiber morphology in the subepithelial and muscular layer. Representative 40 × histological images of Hart’s Elastin stain (**a**) along the circumferential (i, iii, v, vii) and axial (ii, iv, vi, vii) planes for the *Fbln5*^+/+^ (i, ii), *Fbln5*^+/−^ (iii, iv), *Fbln5*^-/-^ with grade 1 POP (v, vi), and *Fbln5*^-/-^ with grade 2/3 POP (vii, viii) mice. Layers of the vagina are denoted as: epithelium (e), subepithelium (s), muscularis (m), and adventitia (a). Elastin area fraction (AF) in the *Fbln5*^+/+^ (black; n = 3), *Fbln5*^+/−^ (grey; n = 3), *Fbln5*^-/-^ with grade 1 POP (light grey; n = 3), and *Fbln5*^-/-^ with grade 2/3 POP (dark grey; n = 3) mice along the circumferential plane (**b**). Fibulin-5 insufficiency and POP significantly affected elastin area fraction within the muscularis (p = 0.001). Elastin AF decreased in the *Fbln5*^−/−^ vagina with grade 1 (p = 0.004) and 2/3 (p = 0.006) POP compared to *Fbln5*^+*/*+^. Elastin AF decreased in the *Fbln5*^−/−^ vagina with grade 1 (p = 0.005) and 2/3 (p = 0.007) POP compared to *Fbln5*^+/−^. Elastin AF along the axial plane (**c**). Fibulin-5 insufficiency and POP significantly (p < 0.001) affected elastin AF within the muscularis and subepithelium. In the muscularis, elastin AF decreased (p < 0.001) in the *Fbln5*^−/−^ with grade 1 and 2/3 POP compared to *Fbln5*^+*/*+^ and *Fbln5*^+/−^. Elastin AF decreased (p = 0.007) in *Fbln5*^+/−^ compared to *Fbln5*^+*/*+^*.* In the subepithelium, elastin AF decreased in the *Fbln5*^−/−^ vagina with grade 1 (p = 0.004) and 2/3 (p = 0.003) POP compared to *Fbln5*^+*/*+^. Elastin AF decreased in the *Fbln5*^−/−^ vagina with grade 1 (p = 0.004) and 2/3 (p = 0.003) POP compared to *Fbln5*^+/−^. Elastic fiber length along the circumferential (**d**) and axial (**e**) planes. Elastic fiber tortuosity along the circumferential (**f**) and axial (**g**) planes. Fibulin-5 insufficiency and POP significantly affected elastic fiber tortuosity within the subepithelium circumferentially (p = 0.03). Elastic fiber tortuosity in the subepithelium increased circumferentially (p = 0.03) in the *Fbln5*^−/−^ vagina with grade 1 POP compared to *Fbln5*^+/−^ and *Fbln5*^−/−^ vagina with grade 2/3 POP. Data is reported as mean ± SEM. Statistical significance is denoted by *p < 0.05, **p < 0.01 and ***p < 0.001.

A one-way ANOVA showed that fibulin-5 insufficiency and POP significantly affected elastin (Fig. 6a) area fraction within the muscularis circumferentially (p = 0.001; Fig. 6b) and axially (p < 0.001; Fig. 6c). Further, fibulin-5 insufficiency and POP significantly affected elastin area fraction within the subepithelium axially (p < 0.001; Fig. 6c). Tukey’s post-hoc test showed that in the muscularis, elastin area fraction decreased circumferentially in the *Fbln5*^−/−^ vagina with grade 1 (p = 0.004) and 2/3 (p = 0.006) POP compared to *Fbln5*^+*/*+^. In the muscularis, elastin area fraction decreased circumferentially in the *Fbln5*^−/−^ vagina with grade 1 (p = 0.005) and 2/3 (p = 0.007) POP compared to *Fbln5*^+/−^. In the muscularis, elastin area fraction decreased axially (p < 0.001) in the *Fbln5*^−/−^ vagina with grade 1 and 2/3 POP compared to *Fbln5*^+*/*+^ and *Fbln5*^+/−^ vaginas. Further, elastin area fraction decreased (p = 0.007) axially in *Fbln5*^+/−^ compared to *Fbln5*^+*/*+^*.* In the subepithelium, elastin area fraction decreased axially in the *Fbln5*^−/−^ vagina with grade 1 (p = 0.004) and 2/3 (p = 0.003) POP compared to *Fbln5*^+*/*+^. In the subepithelium, elastin area fraction decreased axially in the *Fbln5*^−/−^ vagina with grade 1 (p = 0.004) and 2/3 (p = 0.003) POP compared to *Fbln5*^+/−^. Motivated by prior work suggesting that elastic fibers may contribute to SMC contractility^15–18^, a Pearson’s or Spearman’s test evaluated the correlation between contractility and elastin area fraction. Muscularis (ρ = 0.78, p = 0.004) and supepithelium (ρ = 0.86, p < 0.001) elastin area fractions along the axial plane positively correlated with changes in axial stress due to axial contraction (Supplementary Fig. S6). Therefore, as elastin area fraction decreased along the axial plane axial contractility decreased. Fibulin-5 insufficiency and POP did not significantly affect elastic fiber length (Fig. 6d,e). A one-way ANOVA revealed that fibulin-5 insufficiency and POP significantly affected elastic fiber tortuosity within the subepithelium circumferentially (p = 0.03; Fig. 6f). Tukey’s post-hoc test showed that elastic fiber tortuosity in the subepithelium increased circumferentially (p = 0.03) in the *Fbln5*^−/−^ vagina with grade 1 POP compared to *Fbln5*^+/−^ and *Fbln5*^−/−^ vagina with grade 2/3 POP. Fibulin-5 insufficiency and POP did not significantly affect elastic fiber tortuosity along the axial plane (Fig. 6g).

**Figure 7:**
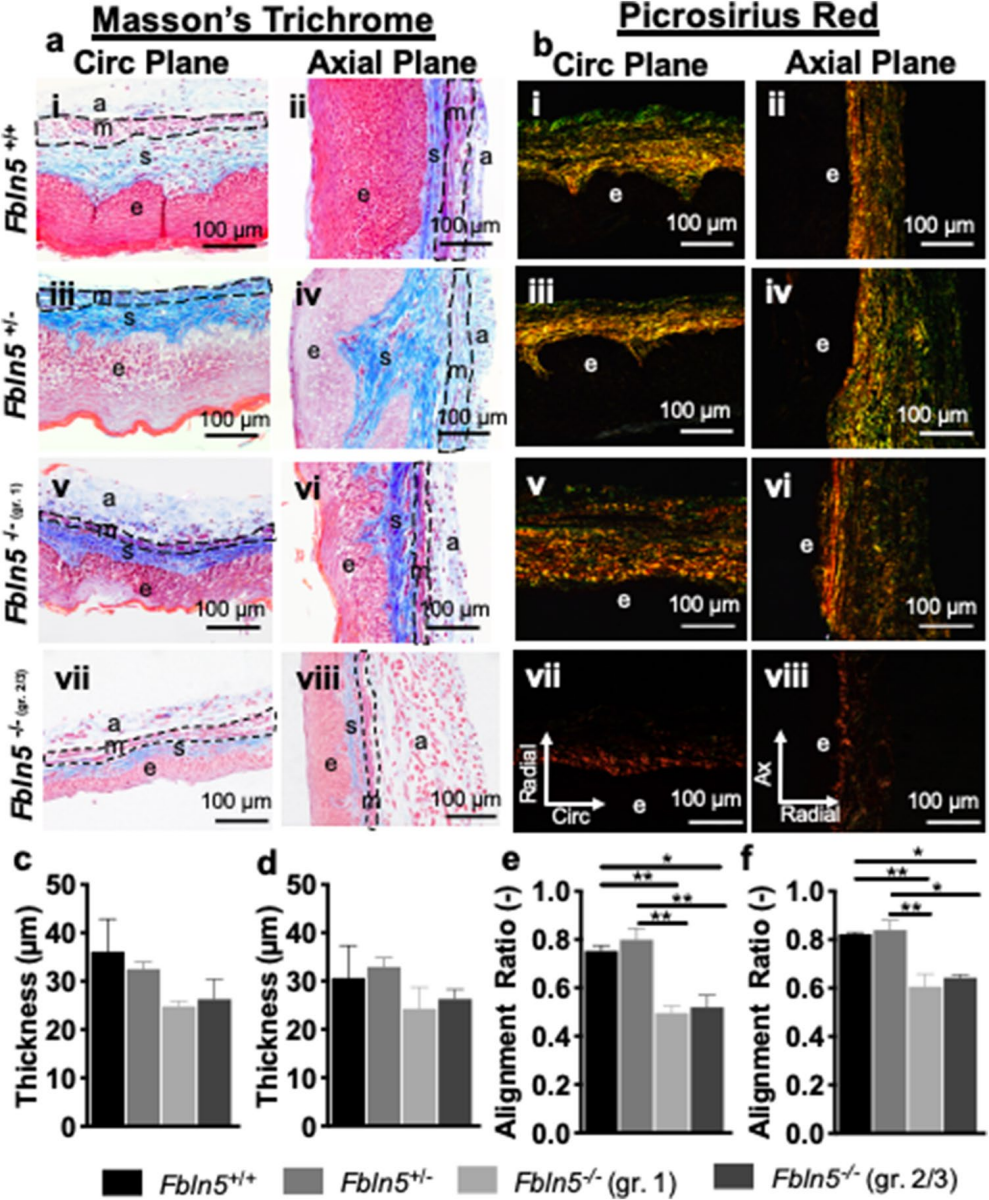
Muscularis thickness and collagen organization. Representative 40 × histological images of Masson’s Trichrome (**a**) and Picrosirius Red (**b**) stains along the circumferential (i, iii, v, vii) and axial (ii, iv, vi, vii) planes in the *Fbln5*^+/+^ (i, ii), *Fbln5*^+/−^ (iii, iv), *Fbln5*^-/-^ with grade 1 POP (v, vi), and *Fbln5*^-/-^ with grade 2/3 POP (vii, viii) mice. The muscular layer is outlined with a black dashed line. Vaginal muscularis thickness is reported along the circumferential (**c**) and axial (**d**) planes for the *Fbln5*^+/+^ (black; n = 3), *Fbln5*^+/−^ (grey; n = 3), *Fbln5*^-/-^ with grade 1 POP (light grey; n = 3), and *Fbln5*^-/-^ with grade 2/3 POP (dark grey; n = 3) mice. Collagen alignment ratio is reported along the circumferential plane (**e**). Fibulin-5 insufficiency and POP significantly affected collagen fiber alignment ratio along the circumferential plane (p < 0.001). The alignment ratio decreased in the *Fbln5*^−/−^ vagina with grade 1 (p = 0.006) and 2/3 (p = 0.01) POP compared to the *Fbln5*^+*/*+^*.* The alignment ratio decreased in the *Fbln5*^−/−^ vagina with grade 1 (p = 0.002) and 2/3 (p = 0.004) POP compared to the *Fbln5*^+/−^*.* Collagen alignment ratio is reported along the axial plane (**f**). Fibulin-5 insufficiency and POP significantly affected collagen fiber alignment ratio along the axial plane (p = 0.002). The alignment ratio decreased in the *Fbln5*^−/−^ vagina with grade 1 (p = 0.008) and 2/3 (p = 0.02) POP compared to the *Fbln5*^+*/*+^*.* The alignment ratio decreased in the *Fbln5*^−/−^ vagina with grade 1 (p = 0.005) and 2/3 (p = 0.01) POP compared to the *Fbln5*^+/−^. Layers of the vagina are denoted as: epithelium (e), subepithelium (s), muscularis (m), and adventitia (a). Data is reported as mean ± SEM. Statistical significance is denoted by *p < 0.05 and **p < 0.01.

Masson’s Trichrome (Fig. 7a) and Picrosirius Red (Fig. 7b) evaluated vaginal muscularis thickness and collagen organization, respectively. Fibulin-5 insufficiency and POP did not significantly affect muscularis thickness (Fig. 7c,d). A one-way ANOVA demonstrated that fibulin-5 insufficiency and POP significantly affected collagen fiber alignment ratio along the circumferential (p < 0.001; Fig. 7e) and axial (p = 0.002; Fig. 7f) planes. Tukey’s post-hoc test revealed that along the circumferential plane the alignment ratio decreased in the *Fbln5*^−/−^ vagina with grade 1 (p = 0.006) and 2/3 (p = 0.01) POP compared to the *Fbln5*^+*/*+^. Along the circumferential plane the alignment ratio decreased in the *Fbln5*^−/−^ vagina with grade 1 (p = 0.002) and 2/3 (p = 0.004) POP compared to the *Fbln5*^+/−^*.* Along the axial plane the alignment ratio decreased in the *Fbln5*^−/−^ vagina with grade 1 (p = 0.008) and 2/3 (p = 0.02) POP compared to the *Fbln5*^+*/*+^*.* Further*,* along the axial plane the alignment ratio decreased in the *Fbln5*^−/−^ vagina with grade 1 (p = 0.005) and 2/3 (p = 0.01) POP compared to the *Fbln5*^+/−^*.*

### Protein expression

**Figure 8:**
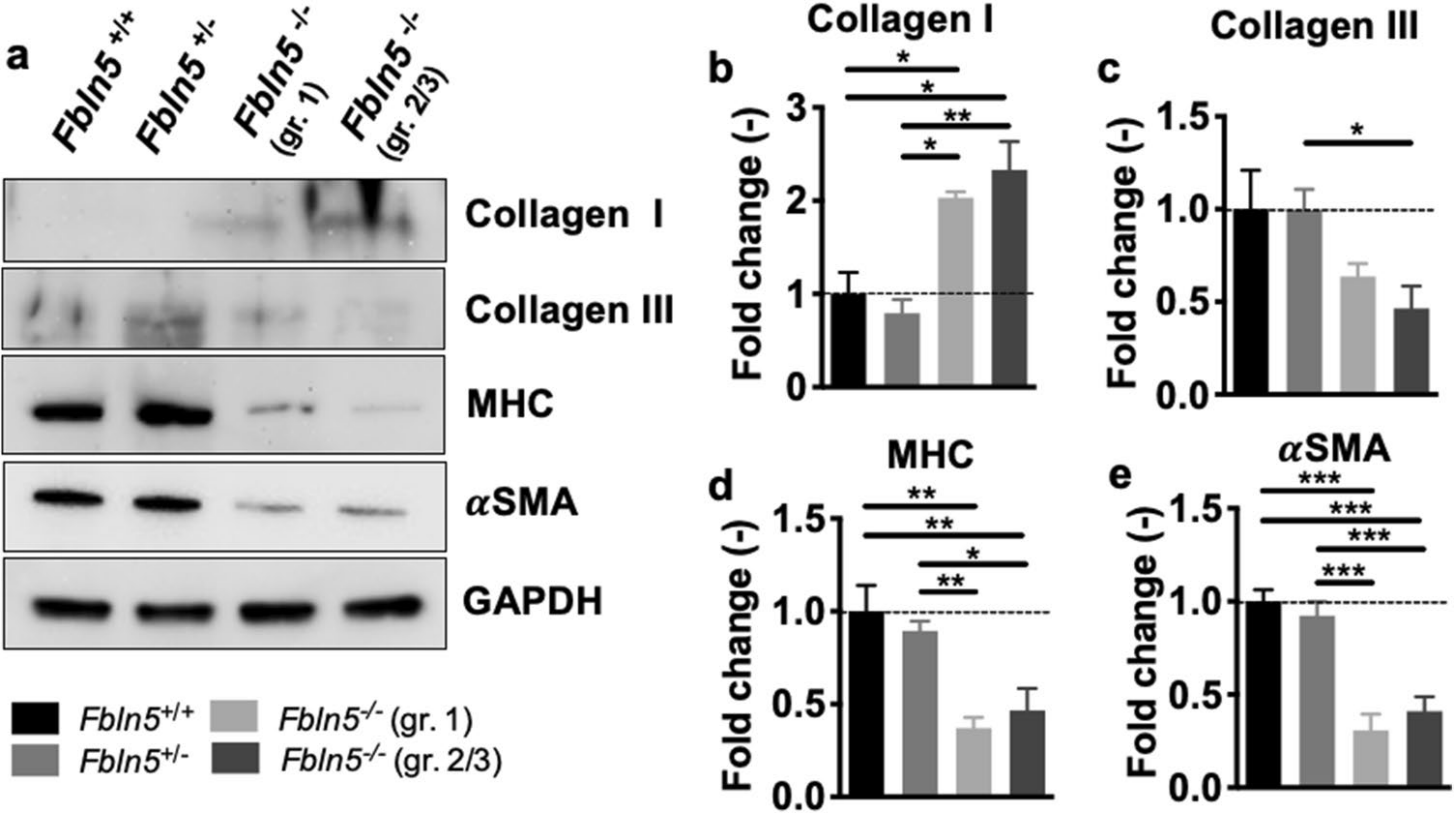
Western blotting analysis for collagens and smooth muscle cell contractile proteins. Representative cropped western blot displaying expression of collagens and smooth muscle proteins (**a**). Cropped blots displayed with full-length western blots reported in Supplementary Figs. S7-13. Densiometric analysis for protein expression of collagen type I (**b**) and collagen type III (**c**) in the *Fbln5*^+/+^ (black; n = 6), *Fbln5*^+/−^ (grey; n = 6), *Fbln5*^-/-^ with grade 1 POP (light grey; n = 6), and *Fbln5*^-/-^ with grade 2/3 POP (dark grey; n = 6) vaginas. Fibulin-5 insufficiency and POP significantly affected collagen type I (p = 0.001) and collagen type III (p = 0.02) protein expression. Collagen type I protein expression increased in the *Fbln5*^−/−^ vagina with grade 1 (p = 0.03) and 2/3 (p = 0.01) POP compared to the *Fbln5*^+*/*+^. Collagen type I protein expression increased in the *Fbln5*^−/−^ vagina with grade 1 (p = 0.01) and 2/3 (p = 0.008) POP compared to the *Fbln5*^+/−^. Collagen type III protein expression decreased in the *Fbln5*^−/−^ vagina with grade 2/3 POP (p = 0.02) compared to the *Fbln5*^+/−^. Densiometric analysis for protein expression of myosin heavy chain (**d**; MHC) and alpha-smooth muscle actin (e; αSMA). Fibulin-5 insufficiency and POP significantly affected (p < 0.001) MHC and αSMA protein expression. MHC protein expression decreased in the *Fbln5*^−/−^ vagina with grade 1 (p = 0.001) and 2/3 (p = 0.006) POP compared to the *Fbln5*^+*/*+^. MHC protein expression decreased in the *Fbln5*^−/−^ vagina with grade 1 (p = 0.006) and 2/3 (p = 0.03) POP compared to the *Fbln5*^+/−^. αSMA protein expression decreased (p < 0.001) in the *Fbln5*^−/−^ vagina with grade 1 and 2/3 POP compared to the *Fbln5*^+*/*+^. αSMA protein expression decreased (p < 0.001) in the *Fbln5*^−/−^ vagina with grade 1 and 2/3 POP compared to the *Fbln5*^+/−^. Protein expression levels were normalized to GAPDH. Data reported as fold change relative to *Fbln5*^+/+^ control. Data is reported as mean ± standard error of mean. Statistical significance is denoted by *p < 0.05, **p < 0.01 and ***p < 0.001.

Western blotting evaluated collagen and smooth muscle proteins expression (Fig. 8a). A nonparametric Kruskal–Wallis test showed that fibulin-5 insufficiency and POP significantly affected collagen type I (p = 0.001; Fig. 8b) and collagen type III (p = 0.02; Fig. 8c) protein expression. Dunn’s post hoc-test showed that collagen type I protein expression increased in the *Fbln5*^−/−^ vagina with grade 1 (p = 0.03) and 2/3 (p = 0.01) POP compared to the *Fbln5*^+*/*+^. Cropped blots are displayed and the full-length blots are shown in Supplementary Figs. S7-13. Collagen type I protein expression increased in the *Fbln5*^−/−^ vagina with grade 1 (p = 0.01) and 2/3 (p = 0.008) POP compared to the *Fbln5*^+/−^. Further, collagen type III protein expression decreased in the *Fbln5*^−/−^ vagina with grade 2/3 POP (p = 0.02) compared to the *Fbln5*^+/−^. A one-way ANOVA demonstrated that fibulin-5 insufficiency and POP significantly affected (p < 0.001) myosin heavy chain (Fig. 8d; MHC) and alpha-smooth muscle actin (Fig. 8e; α SMA) protein expression. Tukey’s post-hoc test showed that MHC protein expression decreased in the *Fbln5*^−/−^ vagina with grade 1 (p = 0.001) and 2/3 (p = 0.006) POP compared to the *Fbln5*^+*/*+^. MHC protein expression decreased in the *Fbln5*^−/−^ vagina with grade 1 (p = 0.006) and 2/3 (p = 0.03) POP compared to the *Fbln5*^+/−^. α SMA protein expression decreased (p < 0.001) in the *Fbln5*^−/−^ vagina with grade 1 and 2/3 POP compared to the *Fbln5*^+*/*+^. α SMA protein expression decreased (p < 0.001) in the *Fbln5*^−/−^ vagina with grade 1and 2/3 POP compared to the *Fbln5*^+/−^. Other comparisons were not statistically significant.

## Discussion

This study assessed vaginal biaxial contractile response and passive biomechanical properties in the fibulin-5 wildtype, haploinsufficient, and deficient mice with POP using an extension-inflation system. Extension-inflation protocols are advantageous biomechanical testing methods due to preservation of vaginal geometry and native SMC-ECM interactions. Further, extension-inflation systems recapitulate aspects of the physiologic environment through axial extension and pressurization. This permitted quantification of vaginal geometry (i.e., outer diameter, length, thickness) and mechanical properties (i.e., stress, stretch, and material stiffness) under physiologically relevant loads. Leveraging extension-inflation methods on the fibulin-5 mouse model was a useful tool to gain understanding of how disrupted elastic fibers altered the biaxial contractile and passive biomechanical function of the vagina and to delineate their potential contribution to POP progression. Fibulin-5 deficiency and POP progression significantly decreased axial contractility, increased vaginal outer diameter, and increased vaginal wall stress.

Vaginal axial contractility decreased in the fibulin-5 deficient vaginas with grade 1 and 2/3 POP. This may be supported by the significant decrease in expression of SMC contractile proteins α SMA and MHC. In human vaginal tissue α SMA area fraction decreased^57,66–68^ and vaginal SMC apoptosis increased with POP compared to non-POP controls^67^. Contrasting the significant decrease in α SMA, Northington et al*.* did not observe significant differences in vaginal axial contraction in response to KCl between POP and controls^69^. The human study investigated vaginal contractility in premenopausal women with stage 0 and I prolapse serving as controls. The prolapse group consisted of premenopausal women with at least stage III prolapse. Including stage I prolapse with the stage 0 as controls may have attributed to not detecting significant differences between the control and prolapsed groups. Further, the women were parous, and it is known that significant vaginal remodeling occurs with pregnancy ^70–72^. The *Fbln5* mice in this study have never been pregnant nor given birth and developed POP independent of these factors.

The decrease in elastin may have contributed to the loss of contractility axially in the fibulin-5 deficient vaginas. Studies suggest that elastic fibers contribute to maintenance of contractile phenotypes of SMC^15–17^. A recent study demonstrated that enzymatically digested elastin significantly decreased axial contractility with no significant changes in circumferential contractility in the C57BL/6 mouse vagina^18^. In this study, elastin area fraction significantly decreased to nearly 0% in the muscularis along the axial plane in the fibulin-5 deficient vaginas with POP. Further, axially contractility decreased as the muscularis elastin area fraction decreased. This suggests that decreased elastin along the axial plane may contribute to decreased vaginal axial contractility. Further, that elastin may be critical for vaginal axial contractility to maintain pelvic organ support. Interestingly, circumferential contractility was maintained for grade 1 POP but significantly decreased back towards the wildtype value as it progressed to grade 2/3 POP. Overall, the fibulin-5 mouse model suggests that elastic fibers may be critical for preserving SMCs and vaginal axial contractility. Further, that altered contractile function may alter SMCs contribution to pelvic organ support. Further, this study demonstrates the need for simultaneously evaluating circumferential and axial vaginal contractility.

The physiologic circumferential and axial stresses significantly increased in the fibulin-5 deficient vagina with grade 2/3 POP and positively correlated with POP grade. Stress was calculated as the force acting over the normal vaginal cross-sectional area due to the intraluminal pressure. Further, vaginal outer diameter increased in the fibulin-5 deficient mice with grade 2/3 POP and positively correlated with POP grade. Likewise, maximum axial stress in prolapsed human vaginal tissue was greater than non-POP controls and positively correlated with POP stage^23^. Further, women with POP presented a larger vaginal outer diameter than non-POP controls^73^. In humans, however, there are no studies to date simultaneously quantifying changes in vaginal geometry and intravaginal pressures. Limited quantitative information exists on changes in pressure with POP in humans^74–76^, but a sensation of an increase in intra-abdominal pressure is a symptom which correlates with increasing stages of POP^2^. Such investigations are necessary to elucidate how these factors alter stress in the human vaginal wall as studies demonstrate that changes in stress induces changes in the structure (i.e., collagen amount and organization) because cells are sensitive to mechanical loads and seek to establish, maintain, or restore a preferred (homeostatic) mechanical environment^77–80^. Future work is needed in vitro and in vivo to evaluate the stress distribution throughout the entire vagina considering its complex geometry (thickness, diameter and length) and how it changes with POP progression^81^. Overall, these findings demonstrate an increase vaginal diameter and wall stress with fibulin-5 deficiency and POP progression. Therefore, calculating stress in the vaginal wall may be valuable when evaluating POP progression to manage and treat POP.

Contrary to the hypothesis, fibulin-5 insufficiency and POP did not significantly affect the material stiffness. The hypothesis that material stiffness would increase in the fibulin-5 haploinsufficient and deficient vaginas with increasing POP grade compared to wildtype controls was motivated by previous work in human vaginal tissue which demonstrated that vaginal material stiffness increased with POP stage^23^. Material stiffness describes a material’s ability to resist changes in deformation, it depends on the underlying microstructure and is independent of changes in geometry. In the fibulin-5 haploinsufficient vagina lack of significant differences in material stiffness compared to the wildtype may be due to no significant changes in collagen protein expression, collagen alignment, and elastic fiber morphology and area fraction (particularly within the subepithelium).

In the fibulin-5 deficient vaginas lack of significant differences in material stiffness compared to the wildtype may be due to remodeling of the collagen fiber network. Collagen type I protein expression increased in the fibulin-5 deficient vaginas with POP compared to the wildtypes, however, collagen type III was not statistically significant. Collagen type I provides greater tensile strength than collagen type III, thus the distribution of the two subtypes plays a critical role in dictating the mechanical properties of soft tissues^82,83^. While collagen type I protein expression increased, collagen fiber alignment decreased (not highly aligned towards preferred direction) in the fibulin-5 deficient vaginas with POP compared to the wildtype control. In addition to collagen subtype content, the organization of the collagen fibers also dictates the mechanical properties of soft tissues. An increase in collagen type I increases material stiffness^84–86^, on the other hand, a decrease in collagen fiber organization decreases material stiffness^87,88^. This may suggest that the interplay between increased collagen type I and decreased organization in the fibulin-5 deficient vaginas contributed to the lack of significant differences in material stiffness compared to the wildtype. To date there are inconsistencies with changes in collagen type I and III in human vaginal tissue with POP^89–92^. This may be due to differences in the methods of analysis, sample location, and the stage of POP across the various studies. Further, there is minimal human data evaluating vaginal collagen organization comparing POP and non-POP controls^93^. Alternatively, previous studies demonstrated that collagen crosslinking played a preferential role over collagen content in dictating material stiffness in the pulmonary artery^94^, patellar tendon^95^, and caudal cruciate ligament^95^. This suggest that in the vagina collagen crosslinking may also be important. Despite increased collagen type I protein expression crosslinks may not be altered, therefore, not significantly affecting material stiffness. Future work is needed quantifying collagen crosslinks in the fibulin-5 mouse model vaginal tissue to support or refute this hypothesis^96^.

Lastly, with fibulin-5 deficiency and POP smooth muscle contractile proteins α SMA and MHC decreased. While MHC is a specific marker for the SMC contractile phenotype and associated with contractility, α SMA is a marker for all SMCs (contractile to synthetic)^97^. The decrease in α SMA expression indicated a decrease in SMCs which may result in altered passive mechanics contributing to the minimal differences observed in material stiffness. SMCs contribute to the passive mechanical behavior of other organs^98^, however, it is unknown how they contribute to the passive mechanical behavior of the vagina. Within vasculature, a study shows that when biochemically decreasing SMC content the passive tissue structurally stiffens and the outer diameter increases^98^. Further, in the lysyl oxidase like-1 mouse model that develops POP with parity, a confluent layer of vaginal SMCs from wildtype controls are materially stiffer than the deficient mice^99^. These studies suggest that SMCs may contribute to vaginal passive mechanical function. This may be dictated by the number of SMCs and their mechanical properties. Future work evaluating the mechanical properties of the fibulin-5 model vaginal SMCs may aid in elucidating their contribution to passive vaginal mechanical function.

The findings in this study contrasted previous findings in fibulin-5 deficient vagina^28^. Herein POP did not significantly affect material stiffness, however, Rahn et al. demonstrated a materially stiffer non-POP control compared to POP^28^. Discrepancies may be due to testing methods and analysis, wherein the previous study conducted a uniaxial ring test, however, this study utilized biaxial extension–inflation protocols. The ring rest consisted of distending the vagina over intervals with 2 min of stress-relaxation until failure, evaluating the engineering stress (force normalized the initial cross-sectional area) and strain behavior^28^. Unlike ring test, biaxial extension-inflation protocols axially stretched the vagina to return it near the physiologic length followed by pressurization about the in vivo measured pressure (not to failure), evaluating the Cauchy stress (force normalized the current cross-sectional area) and stretch behavior. In addition, the previous study used the C3BL/6 J strain as the control, but this study used the similar mixed strain background (C57BL/6 × 129SvEv)^28^. Therefore, the use of different controls may impact the results.

This study was not without limitations. The vagina contains nerves for innervation and the SMCs have receptors for neurotransmitters released by the localized nerves^49,69,100,101^. As a result, SMC contractility can be investigated by direct membrane depolarization with KCl (muscle function), receptor agonist (receptor-dependent function), and nerve stimulation with electric field stimulation (nerve-dependent function). This study evaluated muscle function with KCl which induced a tonic contractile response in the mouse vagina and permitted a consistent analysis across all genotypes and POP grades^18^. Determining and understanding the baseline function with KCl was valuable since receptor- and nerve-mediated responses are normalized to muscle maximum contractile function with KCl^49,50^. Herein, assessment of SMC function independent of receptors and nerves permitted demonstrating elastic fiber potential role in preserving SMC contractile function.

The second limitation is that all fibulin-5 deficient mice in this study displayed at least grade 1 POP by 3 months of age. Therefore, this limited the ability to assess the contractile and passive properties to determine the effect of fibulin-5 deficiency on POP development. The results in fibulin-5 deficient vagina with grade 1 POP suggested that minimal significant changes may be observed in circumferential contractility, vaginal wall stresses, and material stiffness when comparing the fibulin-5 deficient vagina with grade 0 POP to the wildtype control. The fibulin-5 deficient vagina with grade 0 POP was not accessed in this study but future work is needed in a younger cohort of mice to assess fibulin-5 deficiency independent of POP (less than 2 months^44^). A challenge however still remains to assess POP development and progression independent of cofounding factors such as maturation and development, as mice less than 3 months are not fully developed^40^. Despite this, to the authors’ knowledge this is the first study evaluating the progression of POP in the fibulin-5 mouse model. Further, due to pelvic organ descent at the level of the introitus in the grade 2 and 3 POP mice, this presented difficulty in measuring intravaginal pressure in vivo*.* Grade 1 POP pressure measurement was used in grade 2 and 3 mice to determine the physiologic geometry and to calculate the physiologic stresses. In humans, a sensation of increased pelvic pressure is strongly associated with increasing stages of POP^2^. This may imply that a higher POP stage may result in larger pelvic pressure. A larger vaginal pressure in grade 2/3 POP mice may result in a higher vaginal wall stress. Despite being able to calculate stress at the respective in vivo pressure, stress still significantly increased in grade 2/3 POP and positively correlated with POP grade. Therefore, this study still demonstrated that fibulin-5 deficiency and POP progression increased vaginal wall stress. Lastly, the use of a genetically modified model does not permit evaluating spontaneous development and progression of POP as in humans. While this mouse model presented POP with deletion of the fibulin-5 gene various primary effects (e.g., inflammation) may contribute to POP progression^102^. These mice are global knockouts, therefore the *Fbln5* gene is knocked out from birth in all tissues. *Flbn5* also significantly affects the vasculature system^17,103,104^. The vagina contains blood vessel so this systemic factor may also play a role in prolapse progression in this model. Further, the fibulin-5 deficient mice develop POP independent of vaginal birth, which is a primary risk factor for POP in humans. Thus, future work is needed to elucidate the underlying causes of POP progression. This study, however, provided valuable information on changes in vaginal mechanics and microstructure following fibulin-5 deficiency and POP progression to better understand fibulin-5’s contribution to vaginal structure and function.

## Conclusions

It is suggested that elastic fibers play a role in maintaining pelvic organ support and contribute to the pathogenesis of pelvic organ prolapse. Mouse models that are deficient in elastic fiber components, such as fibulin-5, serves as useful tools for understanding elastic fiber interactions with surrounding cells and proteins, and influence on mechanical function. This study multiaxially quantified the microstructure, contractile response, and passive biomechanical behavior to better understand the function of the vagina and changes that occurred with the progression of POP. This study highlighted that fibulin-5 deletion and POP progression decreased vaginal axial contractility and increased vaginal wall stress. Enhanced understanding of cellular and protein interactions’ influence on mechanical function in the development and progression of POP may improve our understanding of the etiology leading to improved preventative and treatment strategies.

## Supplementary Information

Supplementary Information.
