# Supplementary material for: Role of fibulin-5 insufficiency and prolapse progression on murine vaginal biomechanical function
Source: Sci Rep. 2021 Oct 25;11:20956. doi: 10.1038/s41598-021-00351-1 (PMC8546087; doi:10.1038/s41598-021-00351-1)
Supplement: Supplementary file 1 — Supplementary Information. [file 41598_2021_351_MOESM1_ESM.pdf]

Supplementary Material:

Role of Fibulin-5 Insufficiency and Prolapse Progression on Murine Vaginal Biomechanical Function

Gabrielle L. Clark-Patterson[1], Sambit Roy[2], Laurephile Desrosiers[3], Leise R. Knoepp[3], Aritro Sen[2], Kristin S. Miller[1, *]

[1] Tulane University, Department of Biomedical Engineering, New Orleans, 70118, Unites States

[2] Michigan State University, Department of Animal Sciences, Reproductive and Developmental Sciences Program, East Lansing, 48824, Unites States

[3] University of Queensland Ochsner Clinical School, Department of Female Pelvic Medicine and Reconstructive Surgery, New Orleans, 70121, Unites States

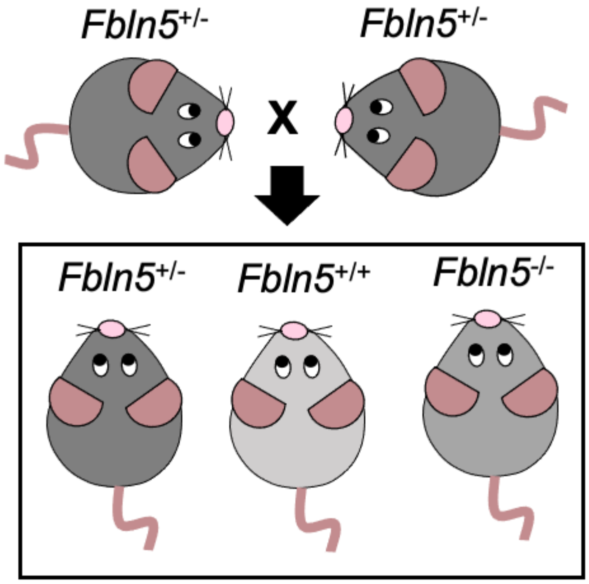

Supplemental Figure S1. Schematic for the generation of fibulin-5 deficient (*Fbln5*$^{-/-}$) mice by crossing a male and female of fibulin-5 haploinsufficient (*Fbln5*$^{+/-}$) mice. This also resulted in the generation of the fibulin-5 wildtype (*Fbln5*$^{+/+}$) and haploinsufficient mice used throughout the study.

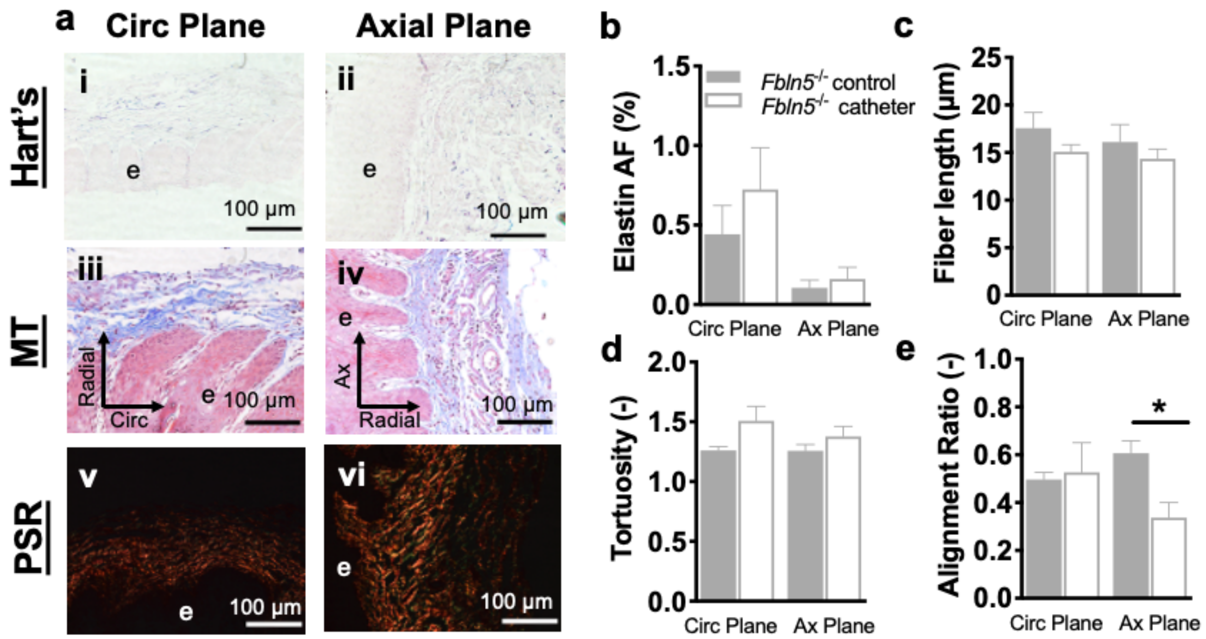

Supplemental Figure S2. Representative Hart's Elastin (a i, ii), Masson's Trichrome (MT; a iii, iv), and Picrosirius Red (PSR; a v, vi) histological section along the circumferential (a i, ii, v) and axial (a ii, iv, vi) planes from the fibulin-5 deficient mice that were balloon catheterized. The layers of the vaginal wall were not well defined. Elastin area fraction (b), elastic fiber length (c), elastic fiber tortuosity (d), and collagen fiber alignment ratio (e) in the fibulin-5 deficient mice that did (n=3; white) and did not (n=3; grey) undergo balloon catheterization. Due to the vaginal wall layers not being well defined in the catheterized mice elastic fiber analysis was not performed by layer. Analysis was performed including the subepithelium, muscularis, and adventitia but excluding the non-load bearing epithelium. This same analysis was performed on the histological sections from the fibulin-5 deficient mice with grade 1 prolapse that did not undergo balloon catheterization. Unpaired t-test did not detect significant differences in elastin area fraction, elastic fiber length, and tortuosity. Collagen fiber alignment decreased in the balloon catheterized mice along the axial plane ($p=0.03$). The epithelium is denoted by "e". Data is reported as mean $\pm$ SEM. Statistical significance is denoted as * $p<0.05$.

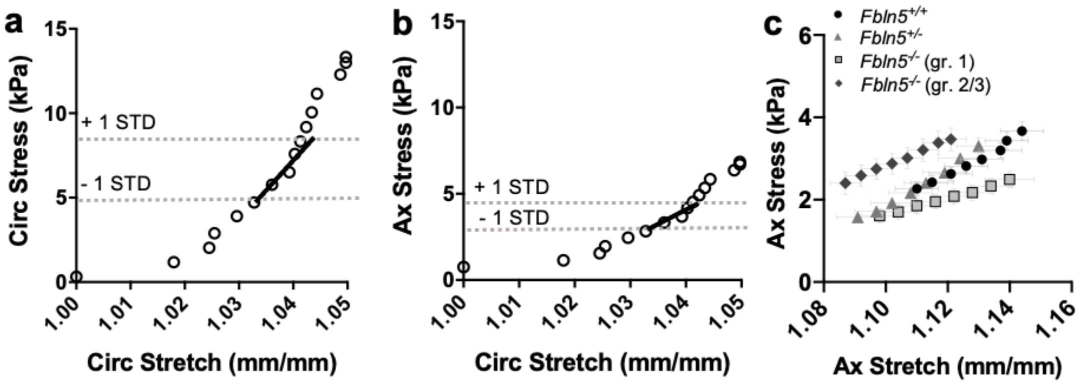

Supplemental Figure S3. Representative circumferential stress versus circumferential stretch curve from pressure-diameter test at the physiologic length (a). Representative axial stress versus circumferential stretch curve from pressure-diameter test at the physiologic length (b). Data analysis calculated stress at every 1 mmHg from 0 to 15 mmHg. The upper stress bound was identified where pressure was 1 standard deviation above the mean (+1STD). The lower stress bound was identified where pressure was 1 standard deviation below the mean (-1STD). In Matlab the polyfit linear function (gray solid line) described the data points within lower and upper stress bounds. The slope of the linear line quantified material stiffness along the circumferential (a) and axial (b) directions. Axial stress versus axial stretch curves for the *Fbln5*$^{+/+}$ (circle; n=13), *Fbln5*$^{+/-}$ (triangle; n=13), *Fbln5*$^{-/-}$ with grade 1 POP (square; n=9), and *Fbln5*$^{-/-}$ with grade 2/3 POP (diamond; n=9) vaginas from force-length test under constant 5 mmHg of pressure (c). This study did not use axial stress versus axial stretch curves to calculate axial material stiffness due to the test being performed under constant pressure. A preliminary analysis calculating material stiffness of the linear region of the stress-stretch curves demonstrated that vaginal axial material stiffness was not significantly different between the axial stress versus axial stretch and axial stress versus circumferential stretch curves, and in good agreement according to a Bland-Altman analysis.[3] The scale is different in figure c and with less data points in order to visualize the data. Data is reported as mean $\pm$ SEM.

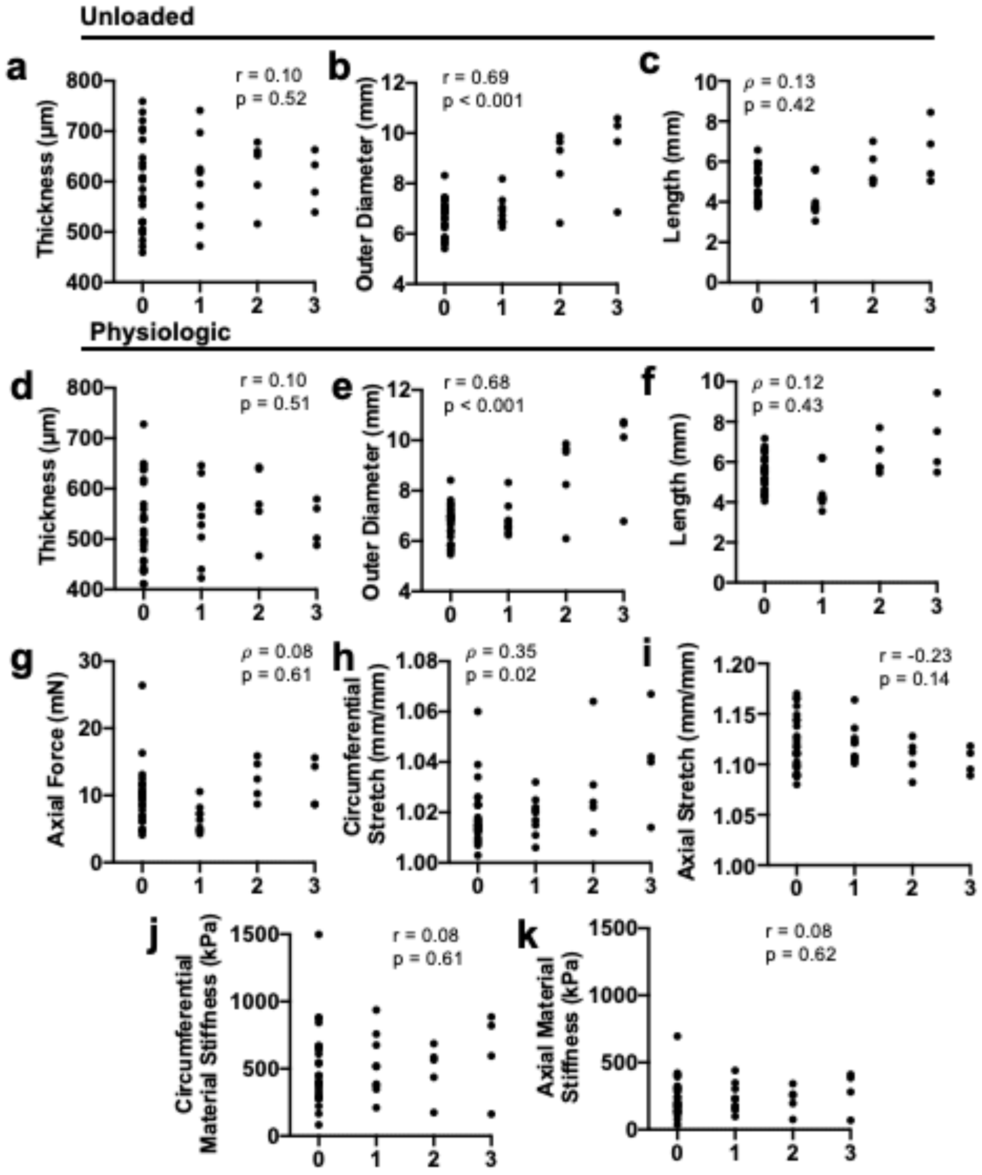

Supplemental Figure S4. Mechanical properties correlated with POP grade. In addition to identifying significant differences in mechanical properties, the data was separated according to POP grade: 0 (n=26), 1 (n=9), 2 (n=5), 3 (n=4). Motivated by prior work demonstrating that the mechanical properties correlated with POP stage in humans[1], a Pearson's or nonparametric Spearman's test evaluated the correlation between passive mechanical properties and POP grade. Unloaded thickness (a), outer diameter (b), and length (c) versus POP grade. A Pearson's correlation demonstrated that the unloaded outer diameter significantly positively correlated with POP grade. Physiologic thickness (d), outer diameter (e), and length (f) versus POP grade. A Pearson's correlation demonstrated the physiologic outer diameter significantly positively correlated with POP grade. Physiologic axial force (g), circumferential stretch (h), and axial stretch (i) versus with POP grade. A Spearman's correlation demonstrated that circumferential stretch significantly weakly positively correlated with POP grade. Circumferential (j) and axial (k) material stiffness versus POP grade. Respective correlation coefficient and p-value are reported in the figure. No other correlations were statistically significant.

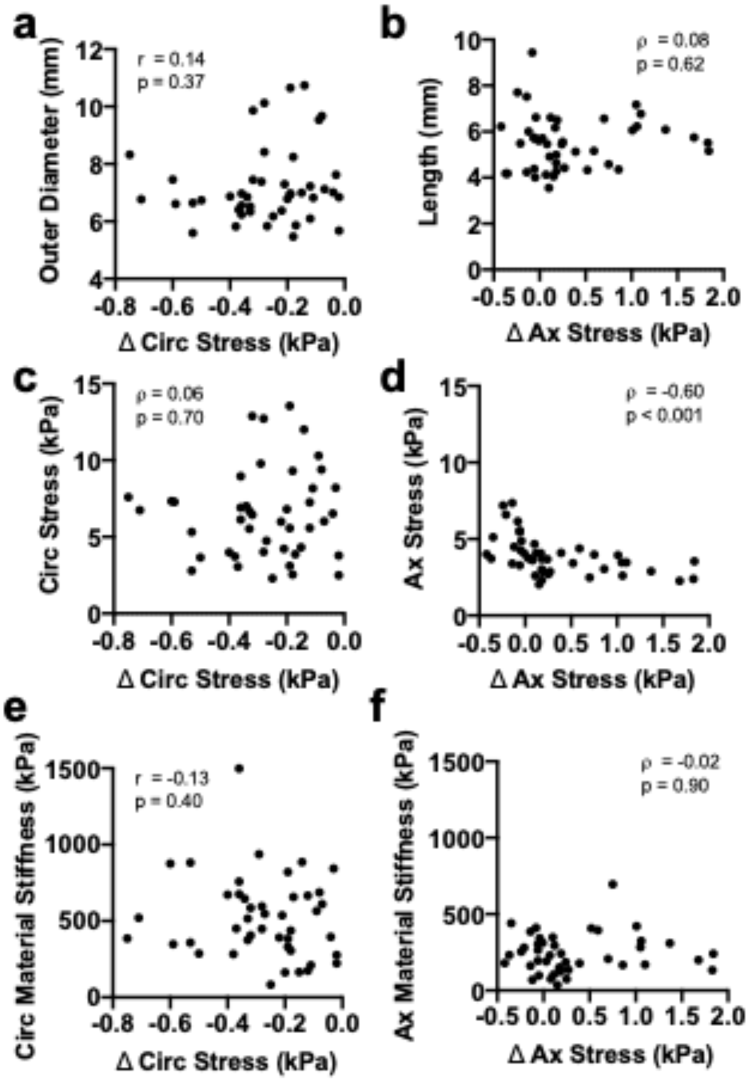

Supplemental Figure S5. Passive mechanical properties correlated with contractility. Motivated by prior work within other tissues demonstrating that the contractile strength correlated with the passive mechanical properties[2], Pearson's and nonparametric Spearman's test evaluated the correlation between passive mechanical properties and contractility. Physiologic outer diameter versus changes in circumferential stress due to circumferential contractility (a). Physiologic length versus changes in axial stress due to axial contractility (b). Physiologic circumferential stress versus changes in circumferential stress due to circumferential contractility (c). Physiologic axial stress versus changes in axial stress due to axial contractility (d). A Spearman's correlation demonstrated that axial stress significantly negatively correlated with changes in axial stress. Circumferential material stiffness versus changes in circumferential stress due to circumferential contractility (e). Axial material stiffness versus changes in axial stress due to axial contractility (f). Respective correlation coefficient and p-value are reported in the figure. No other correlations were statistically significant.

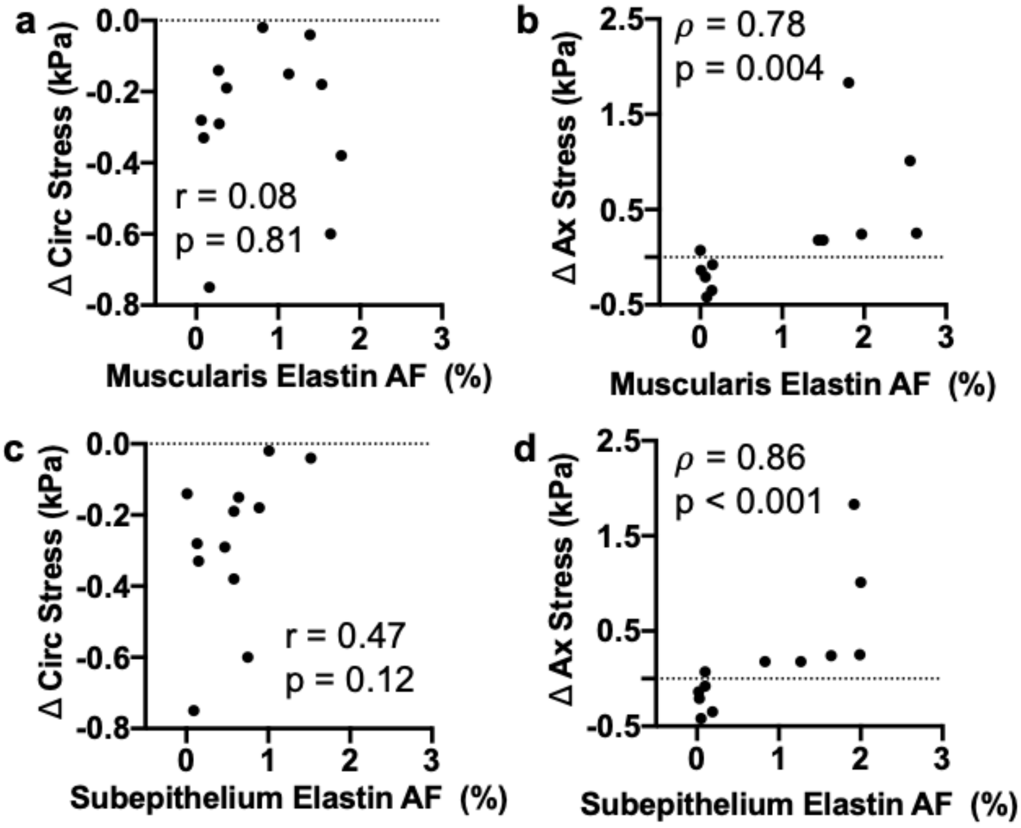

Supplemental Figure S6. Contractility and elastin area fraction correlations. Motivated by prior work suggesting that elastic fibers may be critical to maintain SMC contractility[3-7], Pearson's or Spearman's test evaluated the correlation between contractility and elastin area fraction. Changes in circumferential (circ) stress versus muscularis elastin area fraction (AF) along the circumferential direction (a). Changes in axial (ax) stress versus muscularis elastin area fraction along the axial direction (b). A Spearman's correlation demonstrated that muscularis area fraction along the axial direction significantly positively correlated with changes in axial stress. Changes in circumferential stress versus subepithelium elastin area fraction along the circumferential direction (c). Changes in axial stress versus subepithelium elastin area fraction along the axial direction (d). A Spearman's correlation demonstrated that the subepithelium area fraction along the axial direction significantly positively correlated with changes in axial stress. Respective correlation coefficient and p-value are reported in the figure. No other correlations were statistically significant.

Supplemental Table S1. Passive geometry and mechanical properties at the unloaded and physiologic (pressure and length) configurations.

Genotype (sample size)	*Fbln5*$^{+/+}$ (13)	*Fbln5*$^{+/-}$ (13)	*Fbln5*$^{-/- \text{ grade 1}}$ (9)	*Fbln5*$^{-/- \text{ grade 2/3}}$ (9)	p-value
**Unloaded**					
Thickness (μm)[a]	549 ± 21	631 ± 23	604 ± 28	613 ± 19	0.06
Outer Diameter (mm)[a]	6.93 ± 0.13	6.44 ± 23	6.88 ± 20	9.01 ± 49*, ¥, ^	<0.001
Length (mm)[b]	5.00 ± 0.22	4.73 ± 0.23	4.08 ± 0.30	6.00 ± 0.40^	0.004
**Physiologic**					
Thickness (μm)[a]	478 ± 18	580 ± 21*	538 ± 25	556 ± 21	0.01
Outer Diameter (mm)[a]	6.96 ± 0.13	6.47 ± 0.23	6.84 ± 0.22	9.08 ± 0.56*, ¥, ^	<0.001
Length (mm)[b]	5.64 ± 0.23	5.25 ± 0.25	4.56 ± 0.32	6.63 ± 0.45^	0.004
Axial Force (mN)[b]	7.35 ± 0.71	12.14 ± 1.34*,^	6.51 ± 0.68	12.14 ± 1.03*, ^	<0.001
Circumferential Stretch[b]	1.020 ± 0.004	1.018 ± 0.002	1.019 ± 0.003	1.035 ± 0.013	0.10
Axial Stretch[a]	1.130 ± 0.007	1.110 ± 0.007	1.121 ± 0.007	1.106 ± 0.005	0.06
**Material Stiffness**					
Circumferential (kPa)[a]	639 ± 94	376 ± 47	525 ± 84	546 ± 84	0.09
Axial (kPa)[a]	296 ± 44	174 ± 23	238 ± 40	253 ± 41	0.10

Data reported as mean±standard error of mean.
[a] Derived from a One-way ANOVA
[b] Derived from a Kruskal-Wallis test
$p<0.05$ denoted by * compared to +/+ , ¥ compared to +/-, and ^ compared to -/- with grade 1 POP

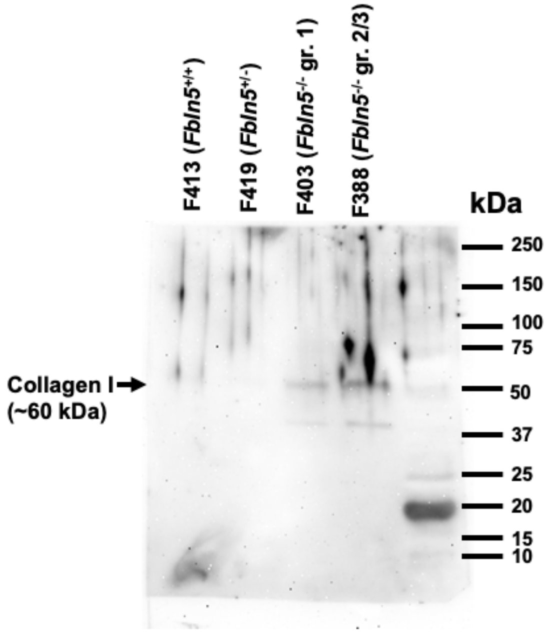

Supplemental Figure S7. Representative full-length western blot for collagen type I.

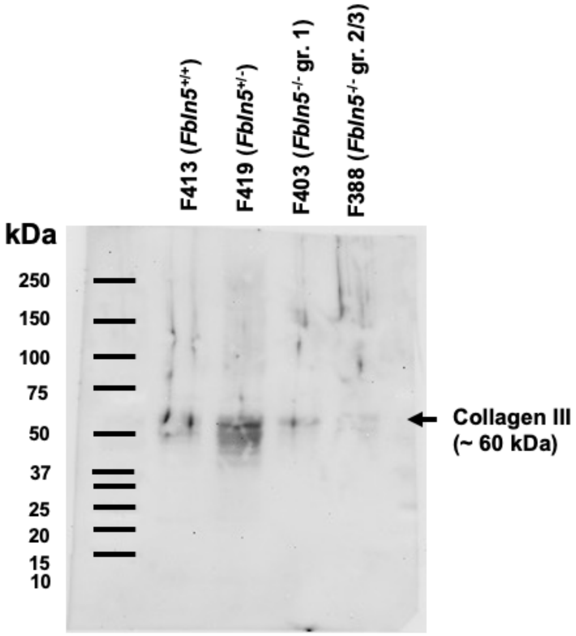

Supplemental Figure S8. Representative full-length western blot for collagen type III.

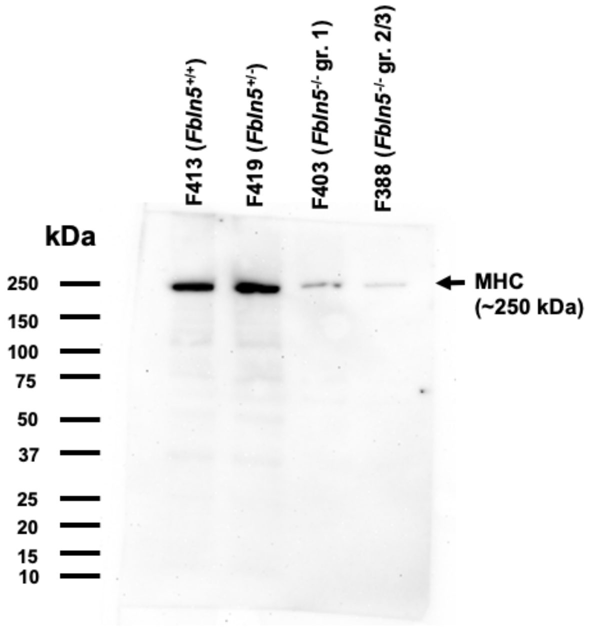

Supplemental Figure S9. Representative full-length western blot for myosin heavy chain (MHC).

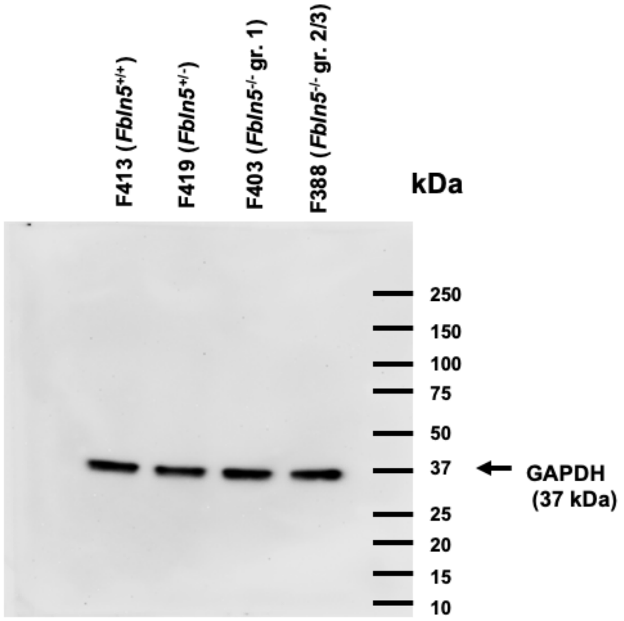

Supplemental Figure S10. Representative full-length western blot for GAPDH.

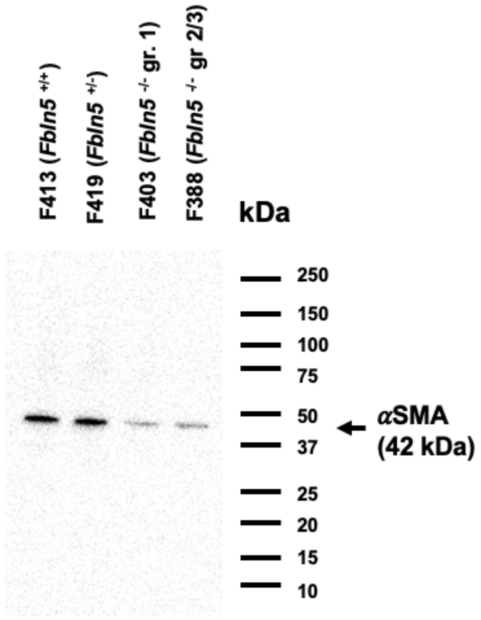

Supplemental Figure S11. Representative full-length western blot for alpha-smooth muscle actin (αSMA) at the lowest exposure time of 0.1 second.

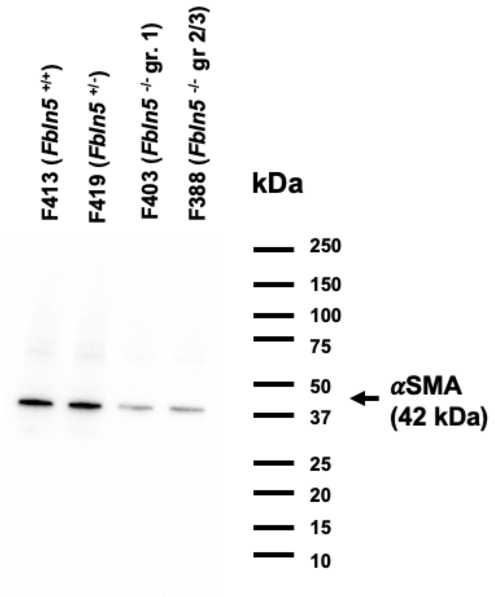

Supplemental Figure S12. Representative full-length western blot for alpha-smooth muscle actin (αSMA) at the optimum exposure time of 1 second.

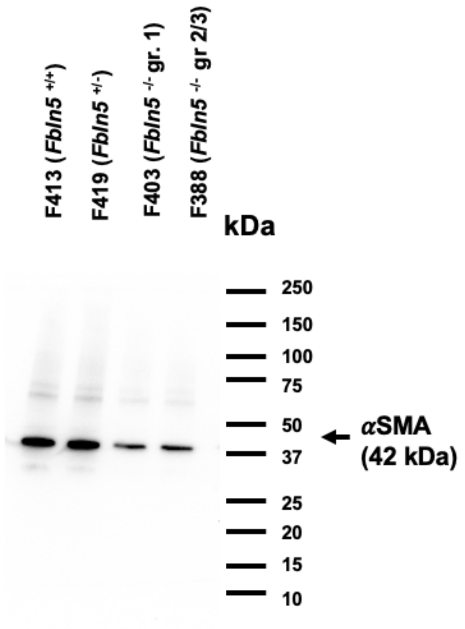

Supplemental Figure S13. Representative full-length western blot for alpha-smooth muscle actin (αSMA) at the highest exposure time of 15 seconds.

References

1 Martins, P. *et al.* Biomechanical Properties of Vaginal Tissue in Women with Pelvic Organ Prolapse. *Gynecologic and Obstetric Investigation* **75**, 85-92, doi:10.1159/000343230 (2013).

2 Murtada, S. I. *et al.* Developmental origins of mechanical homeostasis in the aorta. *Developmental Dynamics*, 11, doi:10.1002/dvdy.283.

3 Davis, E. C. SMOOTH-MUSCLE CELL TO ELASTIC LAMINA CONNECTIONS IN DEVELOPING MOUSE AORTA - ROLE IN AORTIC MEDIAL ORGANIZATION. *Laboratory Investigation* **68**, 89-99 (1993).

4 Karnik, S. K. *et al.* A critical role for elastin signaling in vascular morphogenesis and disease. *Development* **130**, 411-423, doi:10.1242/dev.00223 (2003).

5 Huang, J. B. *et al.* Fibulin-4 Deficiency Results in Ascending Aortic Aneurysms A Potential Link Between Abnormal Smooth Muscle Cell Phenotype and Aneurysm Progression. *Circulation Research* **106**, 583-592, doi:10.1161/circresaha.109.207852 (2010).

6 Murtada, S. I., Lewin, S., Arner, A. & Humphrey, J. D. Adaptation of active tone in the mouse descending thoracic aorta under acute changes in loading. *Biomechanics and Modeling in Mechanobiology* **15**, 579-592, doi:10.1007/s10237-015-0711-z (2016).

7 Murtada, S. I., Ferruzzi, J., Yanagisawa, H. & Humphrey, J. D. Reduced Biaxial Contractility in the Descending Thoracic Aorta of Fibulin-5 Deficient Mice. *Journal of Biomechanical Engineering-Transactions of the Asme* **138**, 7, doi:10.1115/1.4032938 (2016).
